# Multiscale Simulations on the Catalytic Plasticity of CYP76AH1

**DOI:** 10.3389/fchem.2021.689731

**Published:** 2021-06-02

**Authors:** Yufan Qiu, Hongjuan Diao, Ying Zheng, Ruibo Wu

**Affiliations:** ^1^ Key Laboratory of New Drug Design and Evaluation, School of Pharmaceutical Sciences, Sun Yat-sen University, Guangzhou, China; ^2^ Research Center of Integrative Medicine, School of Basic Medical Sciences, Guangzhou University of Chinese Medicine, Guangzhou, China

**Keywords:** cytochrome P450, catalytic promiscuity, molecular dynamic simulations, metadynamics, density functional theory

## Abstract

The catalytic promiscuity and fidelity of cytochrome P450 enzymes are widespread in the skeletal modification of terpenoid natural products and have attracted much attention. CYP76AH1 is involved in key modification reactions in the biosynthetic pathway of tanshinone, a well-known medicinal norditerpenoid. In this work, classical molecular dynamic simulations, metadynamics, and DFT calculations were performed to investigate the protein conformational dynamics, ligand binding poses, and catalytic reaction mechanism in wide-type and mutant CYP76AH1. Our results not only reveal a plausible enzymatic mechanism for mutant CYP76AH1 leading to various products but also provide valuable guidance for rational protein engineering of the CYP76 family.

## Introduction

**FIGURE 1:**
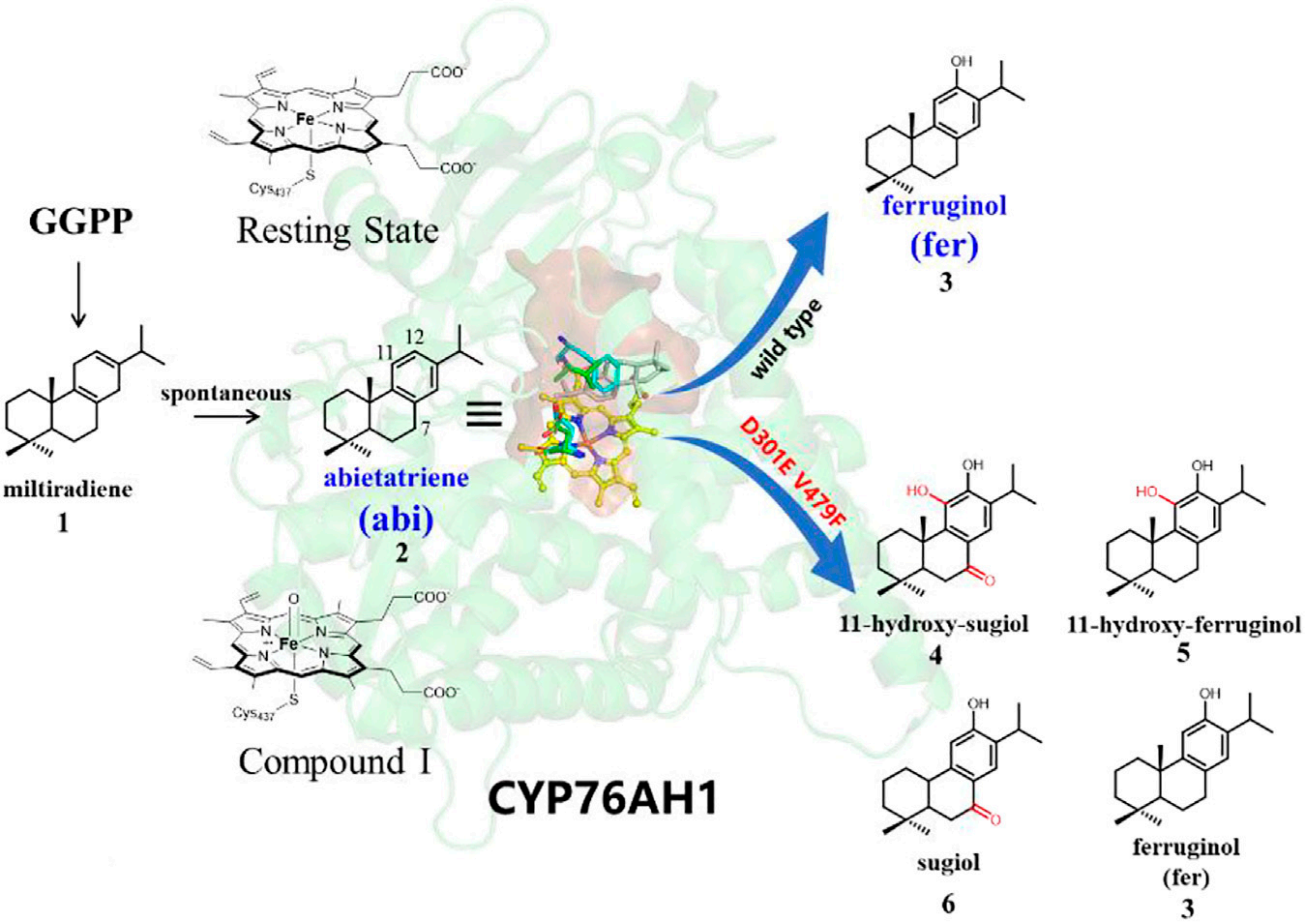
Product fidelity and promiscuity in wild-type and mutant CYP76AH1.

Terpenoids, such as the well-known artemisinin, taxol, tanshinones, and ginsenosides, have received increasing attention for their multiple pharmaceutical values (Yang W. et al., 2020; Zheng et al., 2019). It is well-known that the diverse biological activities of terpenoids are strongly related to the oxidative modification of the cyclic molecular skeletons of terpenoids (Sawai and Saito, 2011). Generally, terpene synthases (TPS) make up the major cyclic carbon skeleton, while its subsequent oxidation reactions of the molecular scaffold are involved by various cytochrome P450s (Bathe and Tissier, 2019). Tanshinones, a kind of abietane norditerpenoid present in the plant *Salvia miltiorrhiza*, have been widely used for the treatment of cerebrovascular and cardiovascular-related diseases (Zhou et al., 2005), and its potential antibacterial, antioxidant, anti-inflammatory, and anticancer biological activities have also been reported (Dong et al., 2011; Robertson et al., 2014). As shown in Figure 1, the cyclization of linear GGPP (geranylgeranyl pyrophosphate) into cyclic miltiradiene is the common step in the biosynthesis of tanshinones (Gao et al., 2009). And P450s are responsible for the subsequent oxidative modification of miltiradiene. So far, several P450s in the CYP76 family were identified to be involved in various oxidative modifications of tanshinones (Guo et al., 2016; Guo et al., 2013). Previous experiments revealed that CYP76AH1 (Guo et al., 2013) could transform miltiradiene to ferruginol via hydroxylation at the C12 position (see Figure 1). It was further demonstrated that the substrate of CYP76AH1 is readily oxidized to the aromatic intermediate abietatriene, which can be spontaneously converted from miltiradiene without enzymatic catalysis (Zi and Peters, 2013). Later, CYP76AH3 was found to be involved in further C7- or (and) C11-oxidation of ferruginol to produce sugiol, 11-hydroxy ferruginol, and 11-hydroxy sugiol (Guo et al., 2016). Recently, it was found that CYP76AH1^D301E,V479F^ efficiently produces a variety of products, including 11-hydroxy sugiol (major), 11-hydroxy ferruginol, sugiol, and ferruginol (minor), with the two mutant residues in the same position as the corresponding positions of CYP76AH3 (Mao et al., 2020), as shown in Supplementary Figure S1, that is, CYP76AH1 would exhibit promiscuous catalytic function through the D301E and V479F double mutations, whereas its wild type produces ferruginol with high fidelity (as summarized in Figure 1).

Regarding the catalytic fidelity and promiscuity of P450s, it has attracted extensive computational and experimental studies. In experimental studies, site-directed mutations have been widely used to identify the plastic residues responsible for fidelity and promiscuity, aiming at more efficient production of desired products (Scheler et al., 2016; Sun et al., 2020; Zhang et al., 2020). In the computational field, various molecular dynamic (MD) simulations, DFT, and QM/MM calculations have been employed to probe key features of P450s, including regioselectivity, stereoselectivity, pocket accessibility, substrate specificity, reactivity, and degree of oxidation (Lonsdale et al., 2013; Guo et al., 2016; Bonomo et al., 2017; Leth et al., 2019; Yang S. et al., 2020). In this study on CYP76AH1, classical and metadynamic MD simulations were performed to examine the differences in pocket dynamics between CYP76AH1 and CYP76AH1^D301E,V479F^, in order to detect the key factors regulating its catalytic fidelity and promiscuity. A rational reaction pathway for the oxidative modification was then proposed and finally demonstrated by DFT calculations. Our results not only reveal the chemical logic of the enzymatic mechanism corresponding to the active pocket plasticity of CYP76AH1 but also provide valuable guidance for rational protein engineering of CYP76AH1 to enhance its catalytic efficiency or selectivity.

## Computational Details

### Preparation of Simulation Systems

The crystal structure of CYP76AH1 (PDB ID: 5YLW) (Gu et al., 2019) was utilized to build eight modeling systems, as summarized in Table1. As well-known, the resting state and Compound I (Cpd I) state (see Figure 1) are two critical states in the P450 catalytic cycle (Meunier et al., 2004; Dubey et al., 2016). The systems with resting state are aiming to analyze the binding pocket and the plausible binding poses of ferruginol. The systems of Compound I state are the reactive states prepared to detect reactivity and possible reaction pathways. Structures of the mutant enzyme and Cpd I were prepared by Maestro of Schrödinger package (version 11.1) (Schrödinger, LLC: New York, NY, 2017). Since the ligand is missing in crystal structures, molecular docking of abietatriene (abi for short) and ferruginol (fer for short) was performed with Glide module. Two molecules were generated and prepared by LigPrep module. The rules for selecting optimal docking pose were defined as below: 1) the distance of Fe-C12 should be less than 5 Å (systems wt/mut-abi-Rest); 2) distance of O-C11 should be less than 4 Å (systems wt/mut-abi-Cpd I); and 3) distance of Fe-C11/C7 should be less than 5 Å (systems mut-fer-C11/C7-Rest). The protonation states of all residues were confirmed by H++ Web server (Gordon et al., 2005), and the pocket residues were further carefully checked.

**TABLE 1:** Illustration of simulation models.

	Type	Substrate	Heme state	System
1	Wild	Apo	Resting state	wt-apo-Rest
2	Mutant	Apo	Resting state	mut-apo-Rest
3	Wild	Abi	Resting state	wt-abi-Rest
4	Mutant	Abi	Resting state	mut-abi-Rest
5	Wild	Abi	Compound I	wt-abi-CpdI
6	Mutant	Abi	Compound I	mut-abi-CpdI
7	Mutant	Fer-C11^a^	Resting state	mut-fer-C11-Rest
8	Mutant	Fer-C7^b^	Resting state	mut-fer-C7-Rest

a Pose that near-attack Fe by C7.

b Pose that near-attack Fe by C11.

### Molecular Dynamics Simulation

Partial atomic charges of substrates were obtained from a restrained electrostatic potential (RESP) method at the HF/6-31G* level with Gaussian09 package (G09 A.01) (Frisch et al., 2009). The General Amber force field (GAFF) (Wang et al., 2005) was used to generate the force field parameters of small-molecule ligands (the abi and fer shown in Figure 1) by leap modules. Partial atomic charge and parameters of heme parameters of the resting state and Cpd I state were both from Shahrokh’s study (Shahrokh et al., 2012). Proteins were described with Amber ff14SB force field (Case et al., 2005). All systems were solvated by cuboid TIP3P (Jorgensen et al., 1983) water boxes with a 10 Å extended distance to the boundary of proteins. Sodium ions were utilized to neutralize all systems.

Three steps of minimization via steepest descent and conjugate gradient methods were taken to prepare the reasonable initial state. First, only water molecules and sodium ions were minimized with a restraint of 100 kcal^−1^ mol Å^−2^. Then only backbone atoms (C, CA, and N) of amino residues were restrained with a smaller force of 50 kcal^−1^ mol Å^−2^, and finally, all atoms were minimized without restraint. Subsequently, systems were heated from 0 to 300 K slowly for 80 ps and then kept at a temperature of 300 K for 20 ps under an NVT ensemble with restraint of 25 kcal^−1^ mol Å^−2^. And additional 300 ps of density equilibration simulations under the NPT ensemble were performed at a constant temperature of 300 K and a pressure of 1.0 atm. At last, all eight systems were implemented MD product and run for at least 400 ns in the NVT ensemble with a time step of 2 fs. Langevin dynamics was used to control the temperature with a collision frequency of 1.0 ps (Izaguirre et al., 2001). Long-range electrostatic interactions were computed with the particle mesh Ewald (PME) method (Essmann and Perera, 1995). Non-bounded cutoff distance was set to 10.0 Å. The SHAKE algorithm (Ryckaert et al., 1977) was applied to constrain hydrogen atoms. All systems were simulated under GPU-accelerated PMEMD program in Amber16 package (Salomon-Ferrer et al., 2013). The MD trajectories were analyzed by several programs including CPPTRAJ (Roe and Cheatham, 2013), Visual Molecular Dynamics (VMD) 1.9.3 (Humphrey et al., 1996), and POVME3.0 (Wagner et al., 2017). The root-measure-square deviations (RMSDs) and root-measure-square fluctuations (RMSFs) involved backbone atoms (C, CA, and N) of proteins were calculated. All the RMSD profiles of eight modeling systems were provided in Supplementary Figure S2. The K-means method was used for the cluster analysis. Principal component analysis (PCA), which recombines the original variables into a new set of unrelated variables to reflect principal information of original variables (Prompers and Bruschweiler, 2002), was performed to analyze the backbone atoms of pocket residues in wt/mut-abi-Rest systems. Pocket volumes of wt/mut-abi-Rest along the MD simulations were calculated by POVME3.0 (Wagner et al., 2017) and provided in Supplementary Figure S3A, and abietatriene was chosen to define the pocket with keyword DefinePocketByLigand, and the parameter GridSpacing was set to 1.0 Å. Pocket shape–based clustering was executed with respect to calculation results of each frame. In total, 800 frames were extracted from 400 ns MD trajectory at regular intervals for calculation of pocket volumes.

Besides, as a powerful technique of enhanced sampling, metadynamics (MTD) (Laio and Parrinello, 2002) was employed in the two systems (mut-abi-C11-Rest and mut-abi-C7-Rest) to map out free energy profiles for estimating the transformation of different ligand binding states, using NAMD 2.15 (Phillips et al., 2005) and Colvars module (Fiorin et al., 2013). The collective variables (CVs) of both systems were set to Fe-C11 distance and Fe-C7 distance, respectively. The Gaussian bias was used to fill the potential energy surface. The hills and widths were set to 0.1 kJ/mol and 0.05 Å, respectively. The Gaussian bias potential was added to the potential energy surface at every 2 ps. A well-tempered algorithm (Barducci et al., 2008) was utilized to decrease the Gaussian hills gradually with a bias factor of 6 to assist free energy profiles converging to definite values. The time step of MTD was set to 2 fs. In total, MTD simulation time of mut-abi-C11-Rest system was 190 and 550 ns for the mut-abi-C7-Rest system.

### DFT Calculations

The reactivity of abietatriene was probed by DFT calculation on cluster models. In detail, the representative structures of cluster analysis from MD simulation trajectory of systems wt-abi-Cpd I and mut-abi-Cpd I were used to generate cluster models (Supplementary Figure S4). The model of Compound I was simplified by removing the side chains of porphyrin. The cysteine connected with porphyrin was replaced by the SCH_3_^-^ group referring to previous modeling (Shaik et al., 2010). Amino residues were truncated with the boundary of α-C atoms which were then saturated with hydrogen atoms. All α-C atoms, hydrogen atoms linked to α-C, and methyl atoms linked to sulfur were frozen during calculations. All DFT calculations were performed with Gaussian 16 package (G16 A.03) (Frisch et al., 2016). All complexes were calculated with doublet states at the level of B3LYP (Lee et al., 1988; Becke, 1993), and empirical dispersion was corrected with D3BJ (Grimme et al., 2010). Geometry optimization and frequency calculations were performed at the B3LYP-D3/def2-SV(P) level (Weigend and Ahlrichs, 2005). The SMD model was applied with water (*ε* = 78.4) (Marenich et al., 2009). The transition-state (TS) structures were searched by Berny algorithm and verified with IRC calculations. Single-point energy calculation was performed at the level of B3LYP-D3/def2-TZVP, and zero-point energy (ZPE) correction was considered. The above theoretical methods and basis set size were used in previously computational studies of P450s (Dubey et al., 2016; Dubey et al., 2017; Su et al., 2019).

## Results and Discussion

### Different Pocket Dynamics of Wild-Type and Mutant CYP76AH1

**FIGURE 2:**
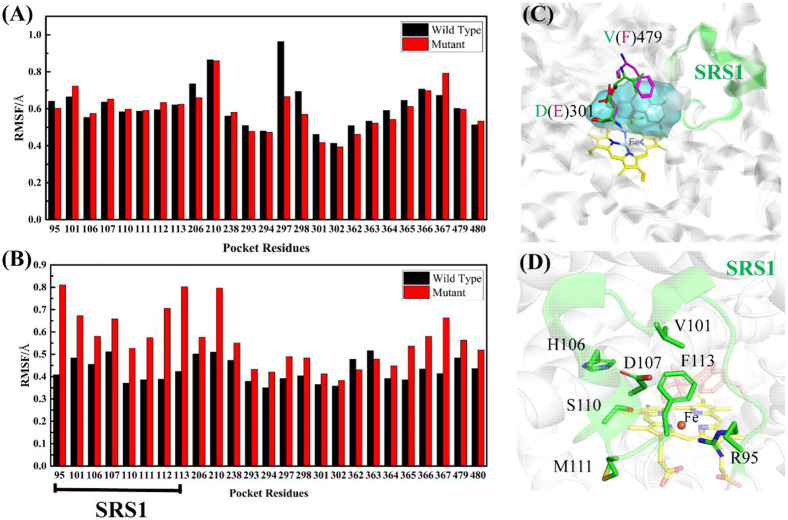
**(A)** Comparison of RMSF values of pocket residues between wt-apo-Rest and mut-apo-Rest systems. **(B)** Comparison of RMSF values of pocket residues between wt-abi-Rest and mut-abi-Rest systems. **(C)** Illustration of the active pocket and the two mutant residues in CYP76AH1. **(D)** Illustration of the flexible SRS1 region and the reactive center Fe.

**FIGURE 3:**
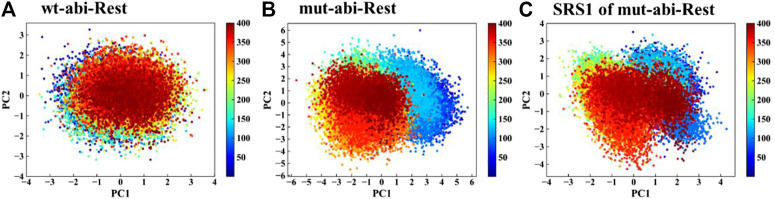
PCA of pocket residues of systems wt-abi-Rest **(A)**, mut-abi-Rest **(B)**, and SRS1 residues of mut-abi-Rest system **(C)**.

The RMSF values of apo-(ligand-free) pocket residues showed minor differences between wild-type and mutant CYP76AH1 (Figure 2A). Differently, the corresponding RMSF values of holo-(abietatriene-bound) pocket residues in CYP76AH1^D301E,V479F^ (see Figure 2B) were much larger, whereas they became smaller in holo-state of wild-type CYP76AH1, suggesting that the active pocket becomes more flexible due to the ligand-induced fit effect and the D301E/V479F double mutant. Regarding the two mutant residues, as shown in Figure 2C, D301 and E301 overlap well, while the conformations of V479 and F479 do not overlap anymore. SRS1 is one of the six well-known substrate recognition sites (SRS) that serve as a key factor for substrate specificity and protein flexibility in previous studies (Gricman et al., 2015). Apparently, here, SRS1 was also found to be the most flexible region of the mutant CYP76AH1 based on the apparent RMSF value (see Figures 2B,D). All these are also confirmed by PCA of the abietatriene-bound active pocket, as shown in Figure 3. The PC1 and PC2 of wild-type system almost overlapped (Figure 3A), while the mutant system appeared to have a scattered distribution, mainly along PC1 (Figure 3B), similar to the PCA of SRS1 (Figure 3C), confirming that the flexibility of SRS1 is enhanced with the binding of the ligand (abietatriene) to the pocket of mutant CYP76AH1 (Figures 2C,D).

**FIGURE 4:**
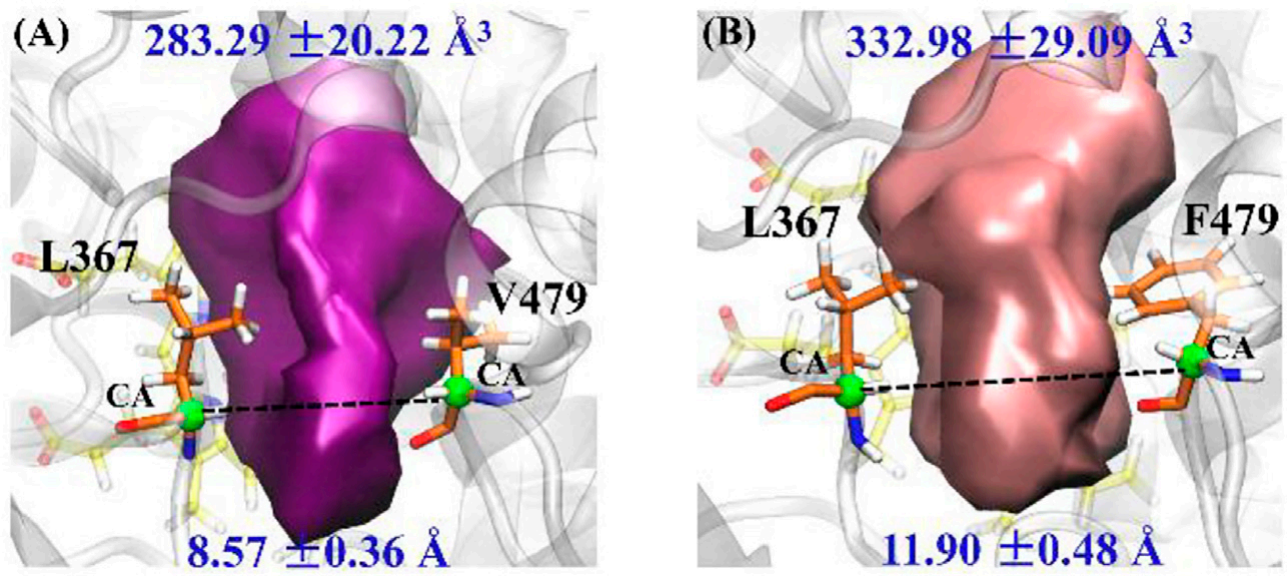
Representative pocket shape of abietatriene-bound CYP76AH1: **(A)** wild type (wt-abi-Rest) and **(B)** the mutant (mut-abi-Rest). The pocket volume and its standard deviation are provided. The distance between two α-C atoms of L367 and V479 (F479 in mutant) is selected to characterize the width of pockets; the details are provided in Supplementary Figure S3B.

Based on pocket volume calculations and further cluster analysis, Figures 4A,B show the most representative active pockets of wild-type and mutant CYP76AH1 bound to abietatriene, respectively. Due to the double mutation (D301E and V479F), the pockets are much larger (showing larger volume and width), bringing enhanced adaptability to different ligands, which is very similar to the pocket analysis in P450s regarding the drug metabolism (Hendrychova et al., 2012). Therefore, we hypothesized that its novel catalytic function to generate multiple products might be due to a change in the pocket shape caused by the D301E/V479F mutant. To determine the exact effect of the mutant, we performed a comprehensive analysis of the binding sites (*see infra*).

### The Induced-Fit Binding Poses Change due to Mutants

**FIGURE 5:**
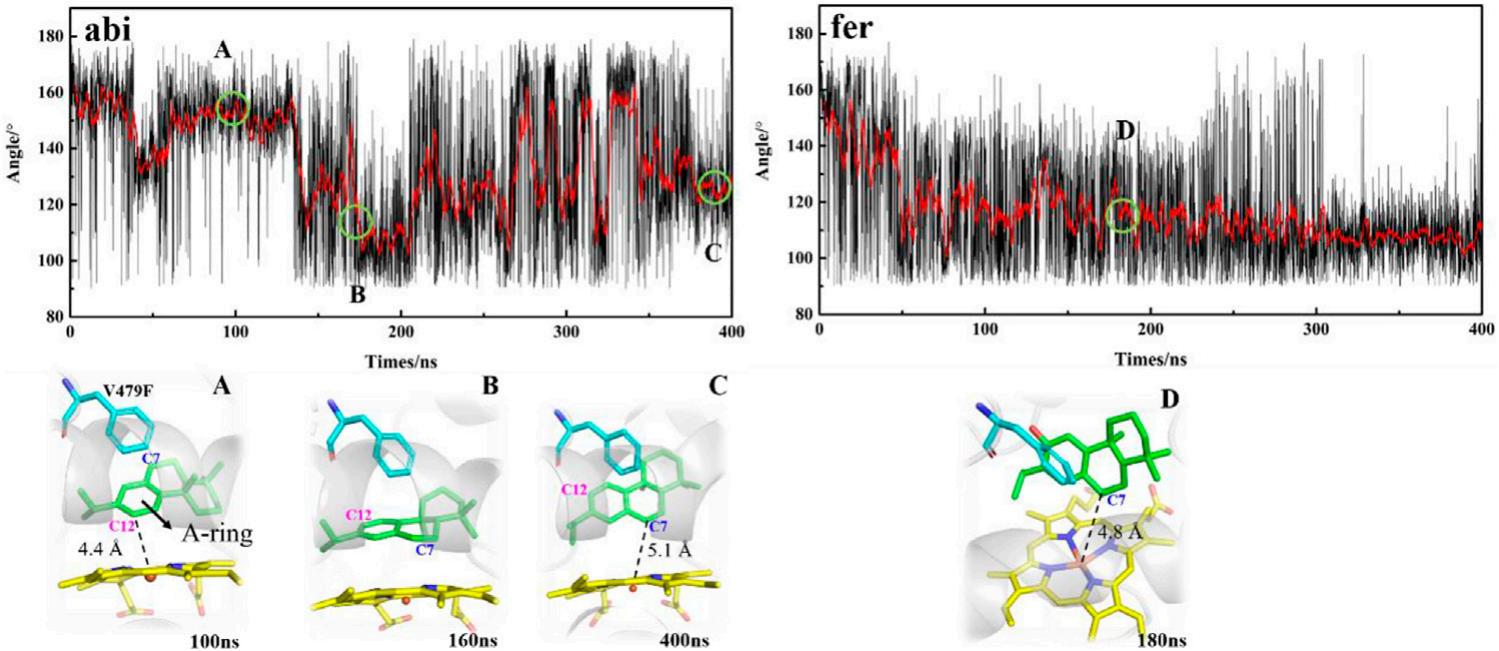
Dihedral angle between A-ring of the ligand and aromatic side chain of F479 along the MD trajectories in mut-abi-Rest and mut-fer-Rest systems. The corresponding representative binding poses are also given. The distance evolutions are supplemented in Supplementary Figures S5, S6.

As shown in Figure 1, in order to decipher the transition from abietatriene (abi) to ferruginol (fer, C12-hydroxy) in the wild type or multiproducts (C7-, C11-, and C12-oxidation) in the mutant, one needs to identify the near-attack binding poses as they trigger oxidative modifications on different carbon atoms. Therefore, MD trajectories for the wt-abi-Rest and mut-abi-Rest systems tracked the evolution of representative binding sites, as summarized in Figure 5. For wild-type CYP76AH1, it implies a potential reactive site on C12 of abietatriene, with a stable Fe-C12 distance of about 5 Å (Supplementary Figure S5A). Interestingly, as shown in Figure 5, not only the C12 site of abietatriene but also an additional reactive site on the C7 site of abietatriene was observed in CYP76AH1^D301E,V479F^. Two attack sites of abietatriene were detected, one tending to Fe-C12 linkage and the other tending to Fe-C7 attack (see Supplementary Figure S5B) for a detailed distance profiles). As can be seen in Figure 5, the first is the C12-near-attack pose (i.e., state A in Figure 5), where the Fe-C12 distance is around 4.4 Å until 130 ns, maintaining an apparent “face-to-face” aromatic stacking interaction between the A ring of abietatriene and the benzene ring of Phe479. Then an intermediate state B was detected by flipping 90°, shifting to an “edge-to-face” aromatic stacking interaction. After that, it attained a new reactive binding pose (i.e., state C in Figure 5) with an Fe-C7 distance of about 5.1 Å. In conclusion, the induced ligand flip and conformational change of Phe479 would result in two reactive binding poses (C12-near-attack and C7-near-attack) in the mutant CYP76AH1, making the modification of the C7 position feasible. It should be noted that the value of the dihedral angle is ranging from 150° to 160° in the A state, and this value becomes more extensive between 100° and 160°, implying that both “face-to-face” and “edge-to-face” aromatic stacking interactions between the A ring and Phe479 would be present in the C state due to ligand flip and dynamic flip of the Phe479 aromatic ring (see Figure 5).

**FIGURE 6:**
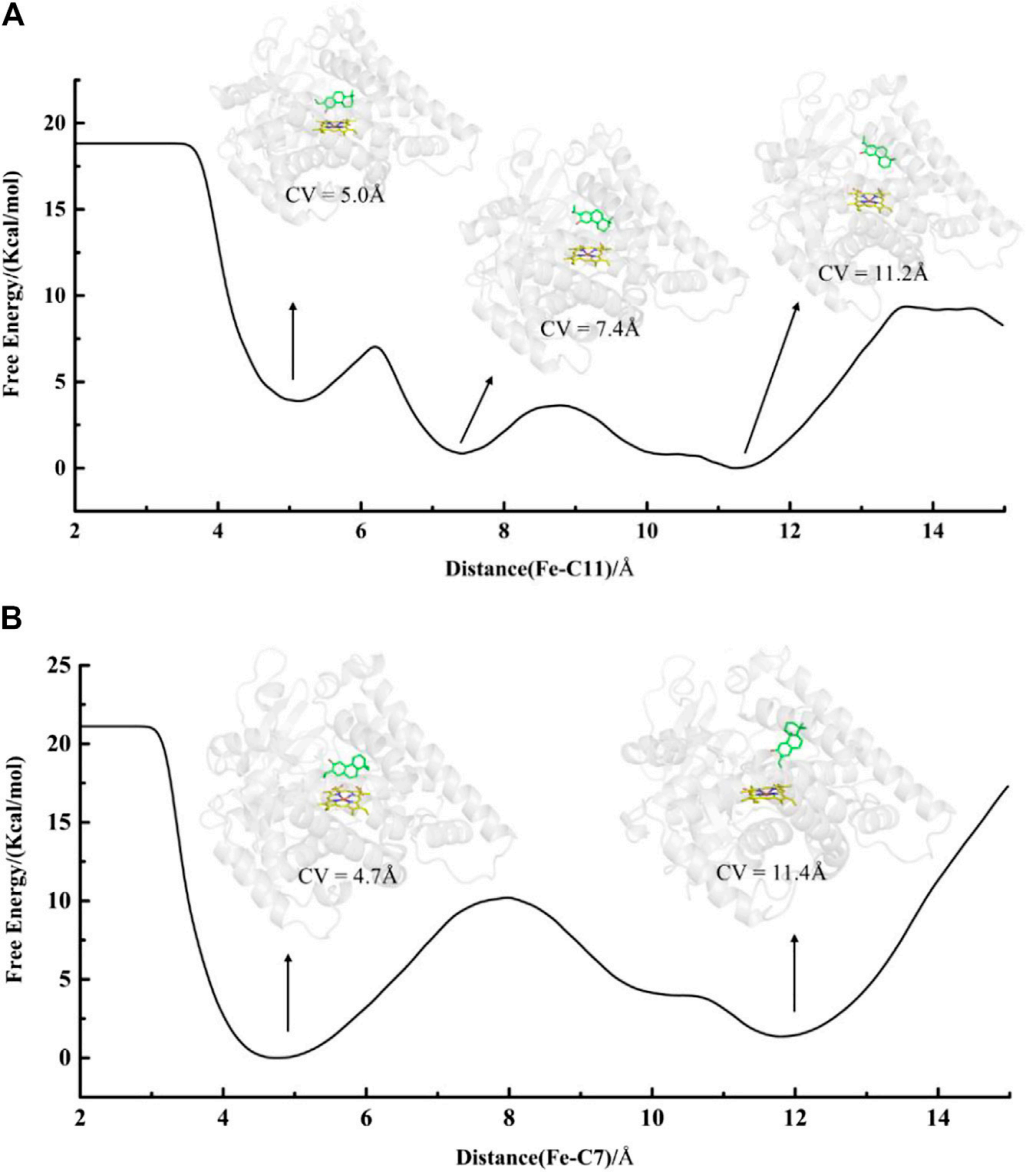
Free energy profiles of binding poses transformation for systems **(A)** mut-fer-C11-Rest and **(B)** mut-fer-C7-Rest. The distance of Fe-C7 and Fe-C11 is defined as CVs which range from 2 to 15 Å.

In addition, an edge-to-face aromatic stacking interaction between the A-ring and Phe479 was also detected in the mut-fer-C7-Rest system (see Figure 5), where ferruginol adopted a stable C7 site near-attack binding pose with a stable Fe-C7 distance of about 4.8 Å (see Supplementary Figure S6A for details). This suggests that C12-hydroxylation from abietatriene to ferruginol may have occurred first in the mutant CYP76AH1, followed by further oxidation on C7, which is consistent with previous experiments that sugiol can be obtained in CYP76AH1^D301E,V479F^. If 11-hydroxy-ferruginol is converted from ferruginol, the C11-near-attack binding pose of ferruginol is required. However, the C11-near-attack binding pose of ferruginol is not present in mutant CYP76AH1, which is confirmed by the expanded distance of Fe-C11 (∼7.5 Å) after 100 ns MD simulations of mut-fer-C11-Rest system (see Supplementary Figure S6B). To further confirm the above analysis of the binding poses, free energy profiles for the plausible binding pose transition were obtained by performing metadynamics simulations (Figure 6). In the mut-fer-C11-Rest system, the C11-near-attack binding pose (CV = 5.0 Å) lies at a metastable minimum, which is thermodynamically and kinetically facile to the other two more stable minimums (CV = 7.4 Å and 11.2 Å). In contrast, in the mut-fer-C7-Rest system, the dominant pose (CV = 4.7 Å) is located in a deep energy basin and is energetically difficult to transfer to the other minimum pose (CV = 11.4 Å).

In conclusion, the predominant binding poses of abietatriene and ferruginol in wild-type and mutant CYP76AH1 were comprehensively determined by detecting the different aromatic stacking patterns seen at the near-attack distance base on our classical MD simulations, and the spontaneous binding pose transition in mutant CYP76AH1 was clearly revealed by MTD simulations. Furthermore, our simulations demonstrate that the V479F mutation is responsible for the induced-fit binding pose transition. We then propose a plausible oxidative modification at the C7 position of abietatriene, which was not clear in previous studies. Furthermore, in the mutant CYP76AH1, C7- and C12(11)-near-attack binding poses were alternatively detected, and their conversion was also captured in the abietatriene bound state. The analysis of all these binding poses explains well the experimental results that the C7-oxidation products are detected in either the V479F single mutant or the D031E/V479F double mutant to CYP76AH1. Considering that only the C7-near-attack binding pose was observed in the mutant CYP76AH1 and no stable C11-near-attack binding pose was observed (see Figure 6), we inferred that all C11-hydroxylation products (see Figure 1) might not be derived from ferruginol but from the precursor abietatriene, which will be further demonstrated by DFT calculations (*see infra*).

#### The Plausible Reaction Pathways of Abietatriene in Wild-Type and Mutant CYP76AH1

**FIGURE 7:**
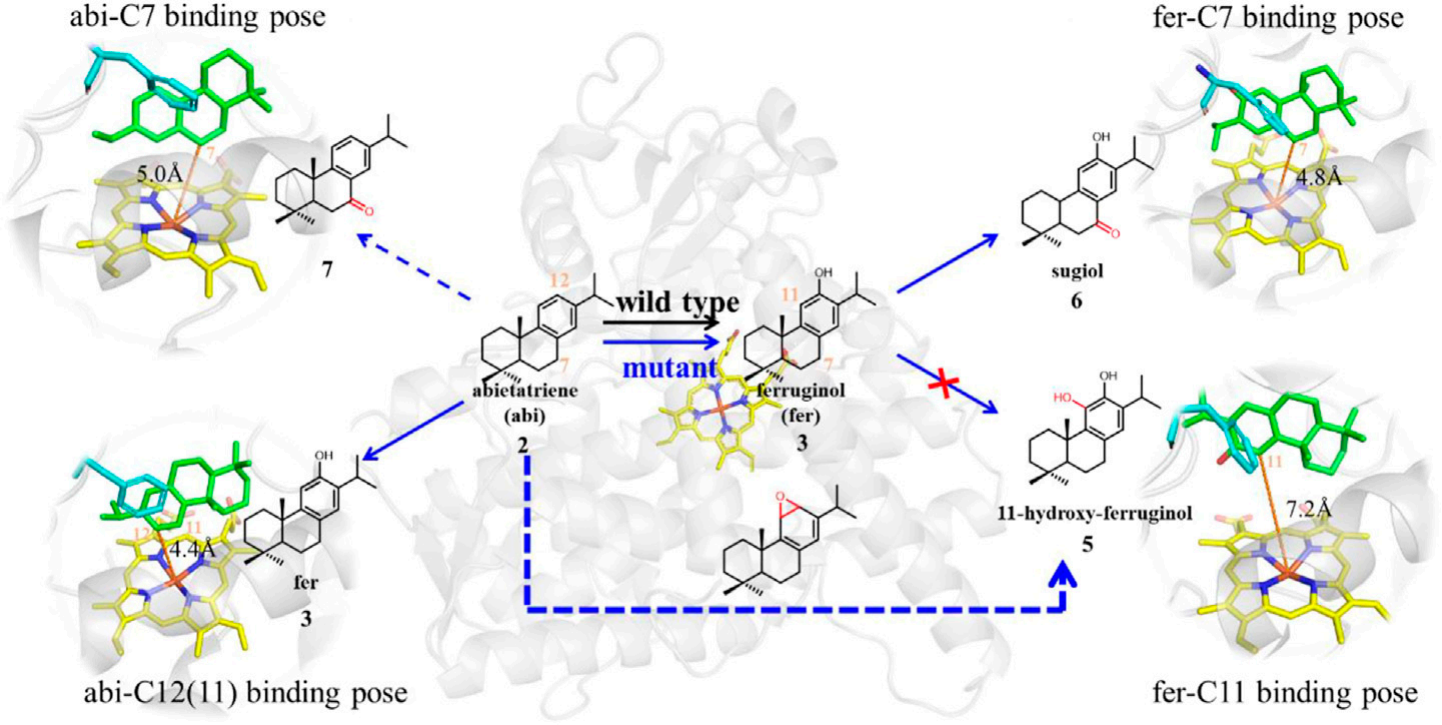
Plausible pathways of CYP76AH1 and CYP76AH1^D301E,V479F^.

Based on the above elaborative analysis of pocket dynamics and ligand (abietatriene and ferruginol) binding poses, we put forward a plausible reaction pathway for wild-type and mutant CYP76AH1 (D301E, V479F) to produce distinctive products, as shown in Figure 7, where both wild-type and mutant enzymes can catalyze the production of ferruginol from abietatriene. Sugiol can be obtained directly from ferruginol, and the formation of 11-hydroxy-ferruginol in this manner is prohibited. In contrast, according to recent studies on the mechanism of 1,2-naphalenediol production from naphthalene, 11-hydroxy-ferruginol may be obtained directly from abietatriene by epoxidation (Bao et al., 2019). Therefore, DFT calculations were performed to probe the reactivity of abietatriene in wild-type and mutant systems based on the truncated protein–ligand structure validated in the aforementioned MD simulations.

**FIGURE 8:**
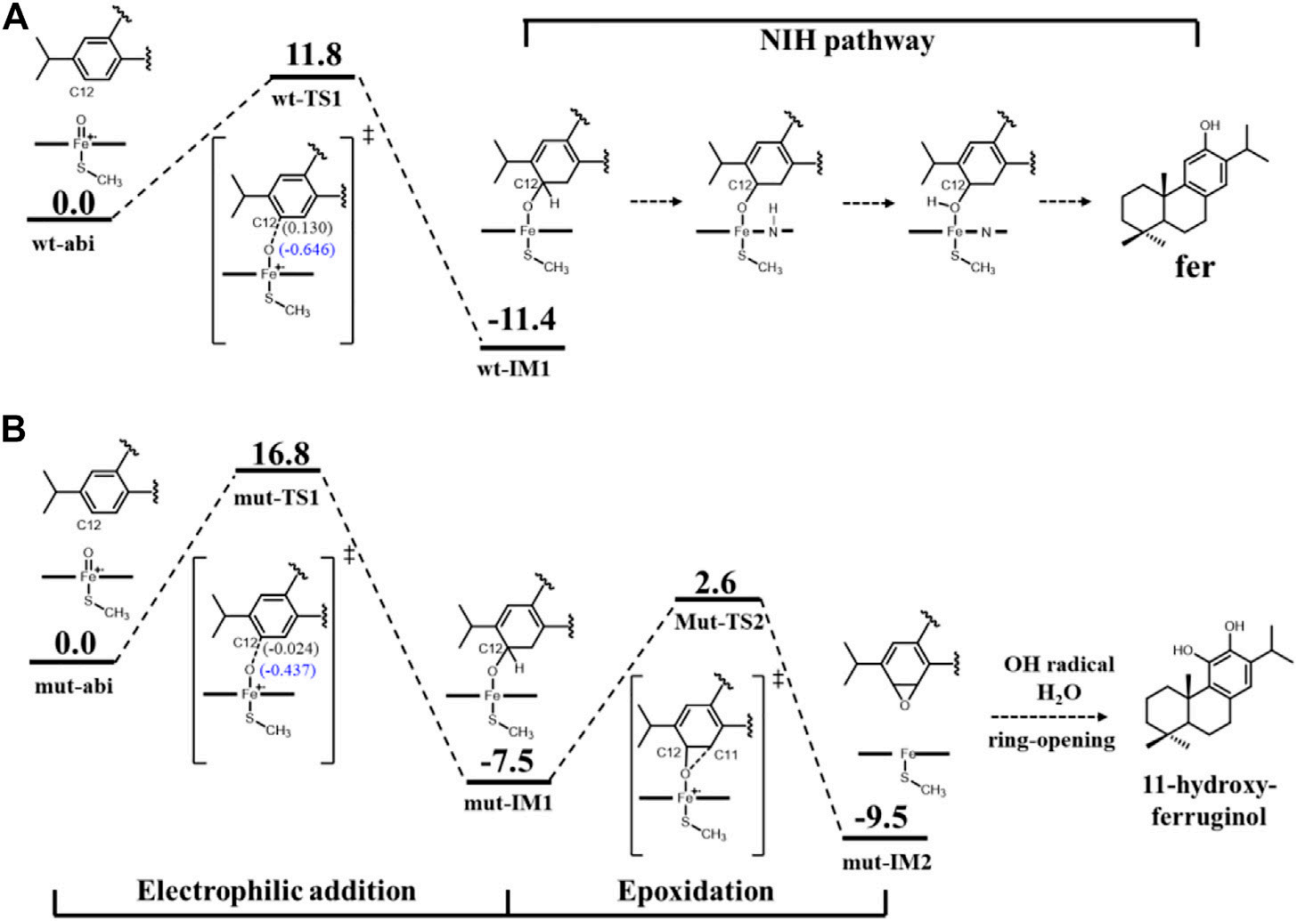
Reaction diagram [B3LYP-D3/def2TZVP//B3LYP-D3/def2SV(P)] for **(A)** wt-abi complex and **(B)** mut-abi complex. The values in brackets represent Mullikan charge. The cluster models are prepared from MD simulations of systems wt-abi-Cpd I and mut-abi-Cpd I as shown in Supplementary Figure S4. The distance of Fe-C12 and Fe-O-C12 angles is reasonable, since the distance is less than 4 Å, and the angle is fluctuating between 110° and 130° (Supplementary Figure S7). The corresponding optimized structures are given in Figure 9.

**FIGURE 9:**
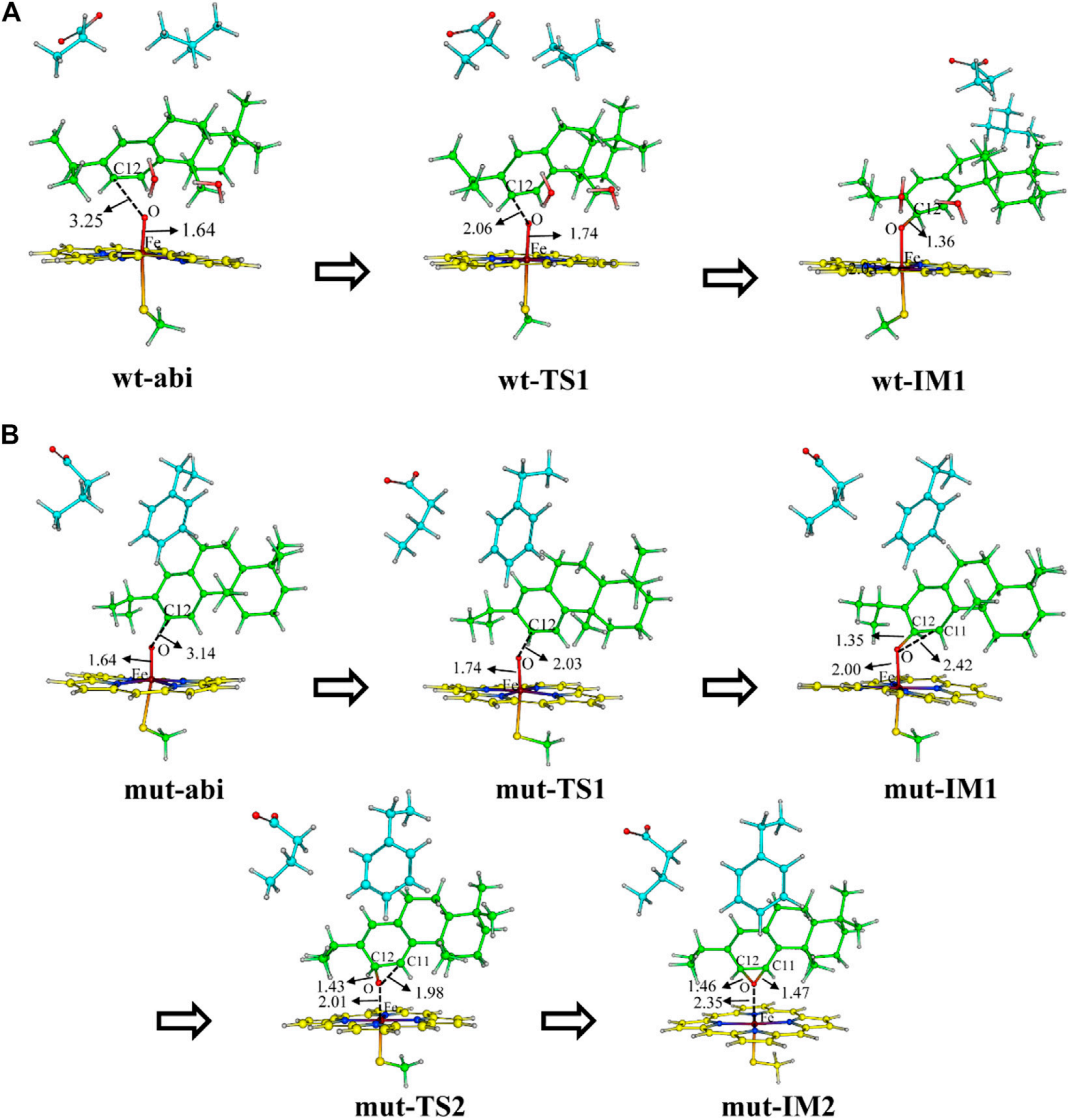
DFT optimized structures of along reaction for the two systems **(A)** wt-abi and **(B)** mut-abi.

First, Figure 8 shows the electrophilic addition reaction of abietatriene, which is the rate-limiting step for aromatic oxidation. The energy barrier of 11.8 kcal/mol for the wild type is much lower than that of the mutant type, which has a reaction barrier of 16.8 kcal/mol. Considering the C12-near-attack binding poses of abietatriene and the critical distances (O-C12, Fe-O) and angles (Fe-O-C12) of the two transition state structures (wt-TS1 and mut-TS1) are very similar (Figures 9A,B), we deduce that the lower reaction barrier of the wild type may be due to the presence of two additional water molecules around the oxygen in the Cpd I reaction center (see Supplementary Figure S4 for details), whereas in our long-time MD simulations, the mutant model did not detect water molecules in this region because the larger side chain of Phe479 prevents water molecules from entering the pocket. According to previous studies (Altun et al., 2006a; Altun et al., 2006b; Kumar et al., 2011), water molecules near the oxygen of Cpd I stabilize the transition state by forming H-bonds and increasing the negative charge of the oxygen. This is consistent with our Mullikan charge prediction (see Figure 8), where the charge of oxygen in wt-TS1 (−0.646) is more negative than that in mut-TS1 (−0.437).

The electrophilic addition reaction would then lead to a tetrahedral σ-complex (IM1 in Figure 8). Considering the energetically favorable character of the conversion from abietatriene to wt-IM1 in the wild type, ferruginol can be further efficiently and specifically yielded through a well-established NIH pathway (see Figure 8) (de Visser and Shaik, 2003). For the mutant system, the electrophilic addition reaction barrier becomes higher (16.8 kcal/mol) but is still kinetically reasonable, and the mut-IM1 can be yielded in view of the release heat (−7.5 kcal/mol). Interestingly, as shown in Supplementary Figure S1, the mutated residues (D301, F479) are indeed conserved and located at the same site of CYP76AH3, which also produces ferruginol but with a lower catalytic efficiency than wild-type CYP76AH1 (Mao et al., 2020). In this sense, these computational results explain the experimental results on the formation of the minor product ferruginol in the mutant CYP76AH1.

Finally, regarding the generation of C11-hydroxy products in the mutant enzyme, the reaction pathway from ferruginol was ruled out since ferruginol maintains an unfavorable attack distance (greater than 7 Å, i.e., fer-C11 binding pose), as shown in Figure 7. Here, we refer to a previously established reaction mechanism (Bao et al., 2019) that naphthalene can be converted to 1,2-naphalenediol, a double ortho-hydroxy product similar to 11-hydroxyl ferruginol, via epoxidation and then opening by OH radicals and water molecules (see Supplementary Figure S8 for details). Since a reactive C12-near-attack binding pose of abietatriene was detected in our MD simulations (provided in Figure 7), we propose that oxygen will attack C11 to form the epoxidation product (mut-IM2). As shown in Figure 8B, DFT calculations show that this process is very facile with a low activation barrier (10.1 kcal/mol) and heat release (2 kcal/mol). Then mut-IM2 can be converted to the final product 11-hydroxy-ferruginol via a ring-opening reaction according to a previous study (Bao et al., 2019), as shown in Figure 8B, Supplementary Figure S8.

## Conclusion

In this work, the catalytic plasticity of the CYP76AH1 enzyme was investigated by multiscale simulations. The reasons for the different reaction pathways of the wild-type and D301E/V479F mutants were revealed, with the former being highly specific for a single product and the latter being very promiscuous for multiple products. It was found that the product promiscuity of mutant CYP76AH1 was mainly attributed to the presence of an alternative (*C12-vs* C7-near-attack) binding pose for the key intermediate abietatriene, whereas a unique C12-binding pose for abietatriene exists in the wild-type enzyme. Meanwhile, the strong pocket plasticity of CYP76AH1 was confirmed by highly adaptive pocket dynamics in response to ligand-induced conformational changes, which was also observed in many P450s, especially those involved in drug metabolism. All these findings provide clues not only for understanding oxidative modifications of tanshinone-like norditerpenoid natural products but also for redesigning CYP76 family enzyme function.

## Data Availability

The original contributions presented in the study are included in the article/Supplementary Material; further inquiries can be directed to the corresponding author.
