# Supplementary material for: Multiscale Simulations on the Catalytic Plasticity of CYP76AH1
Source: Front Chem. 2021 Jun 2;9:689731. doi: 10.3389/fchem.2021.689731 (PMC8207200; doi:10.3389/fchem.2021.689731)
Supplement: Supplementary file 1 [file DataSheet1.docx]

Supplementary Material


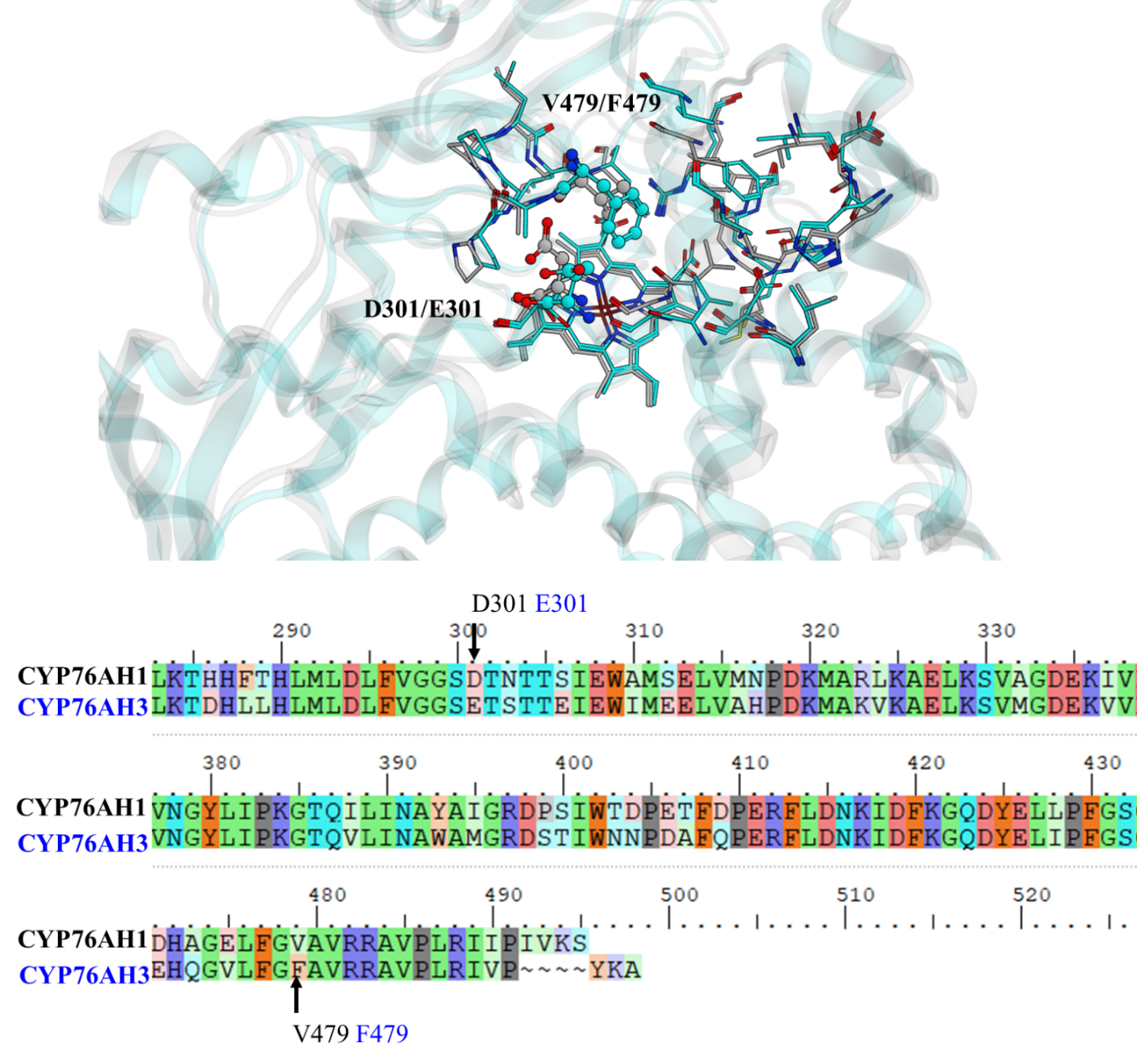


**Figure S1.** Sequence and pocket alignment of CYP76AH1 and CYP76AH3. For protein structure, CYP76AH1 is grey and CYP76AH3 is blue. Key residues of D301/E301 and V479/F479 are represented with ball and stick.


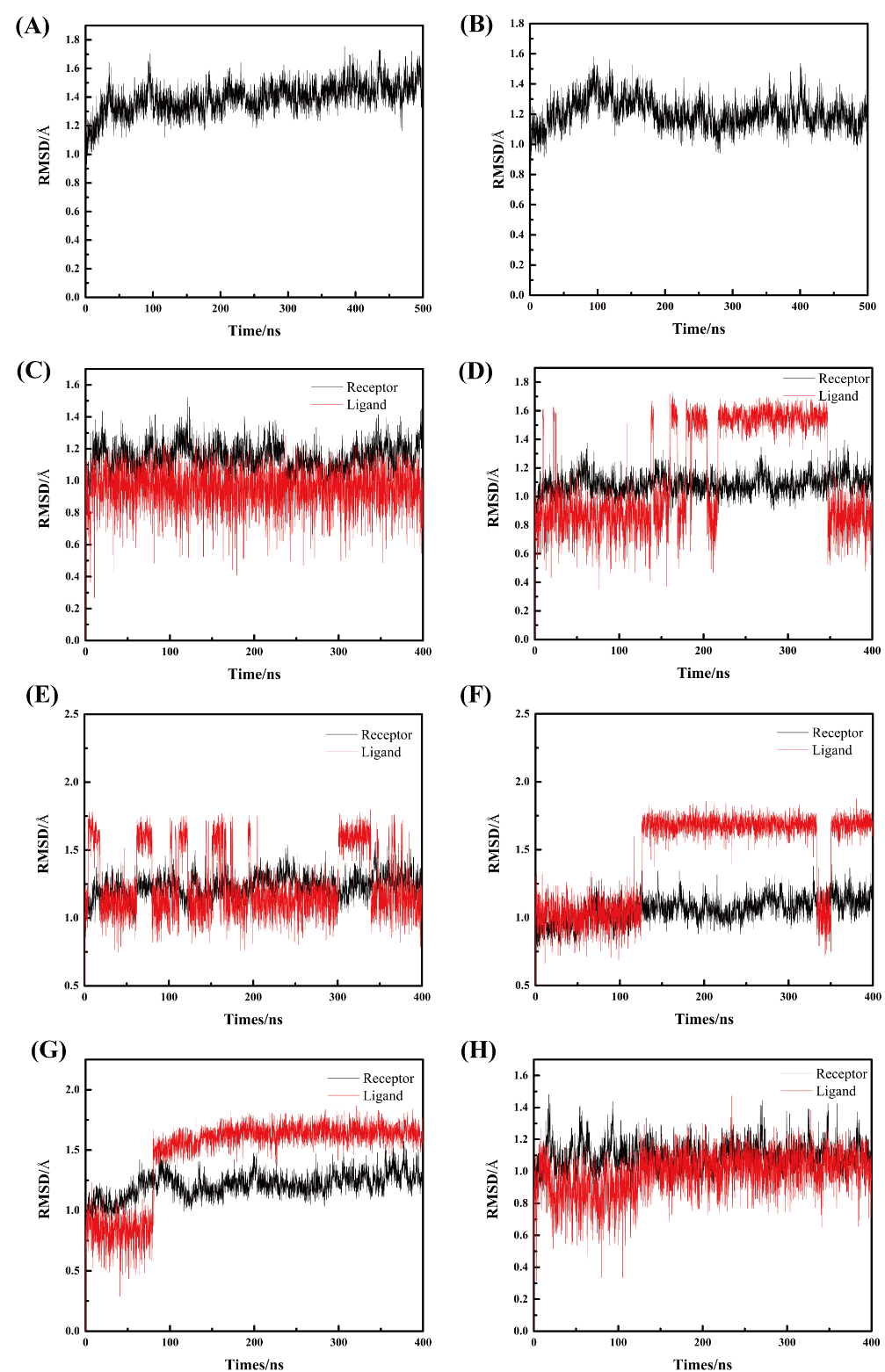


**Figure S2.** RMSD values of systems (A) wt-apo-Rest, (B) mut-apo-Rest, (C) wt-abi-Rest, (D) mut-abi-Rest, (E) wt-abi-Cpd I, (F) mut-abi-Cpd I, (G) mut-fer-C11-Rest and (H) mut-fer-C7-Rest.


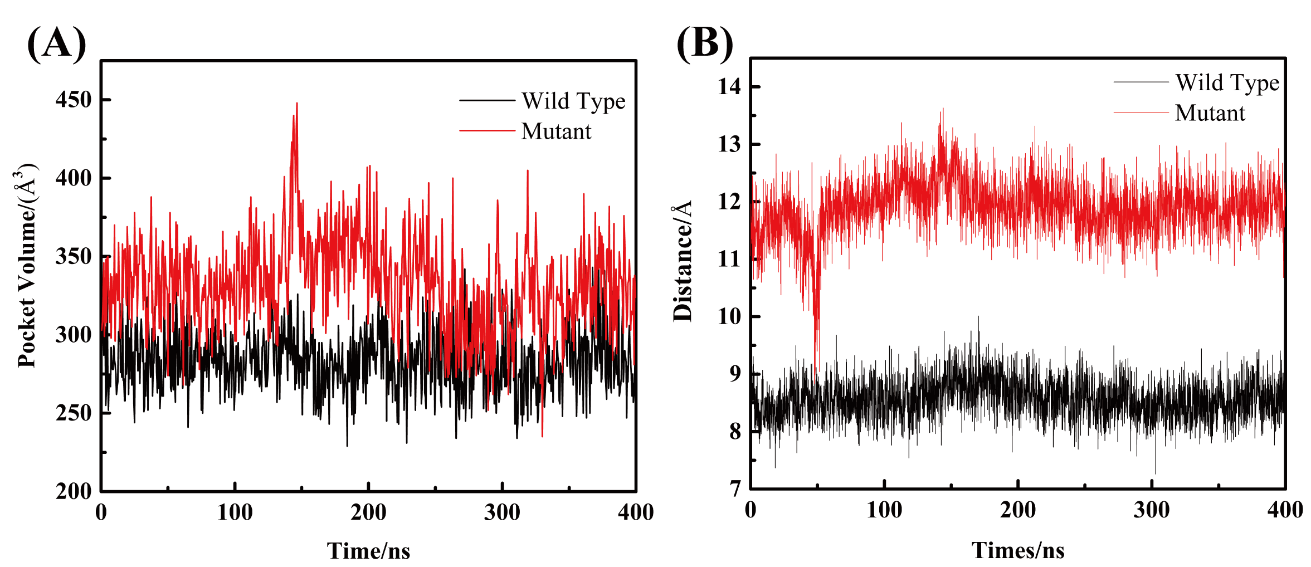


**Figure S3.** Statistics in distances of (A) pocket volumes and two (B) α-C distances with respect to times.


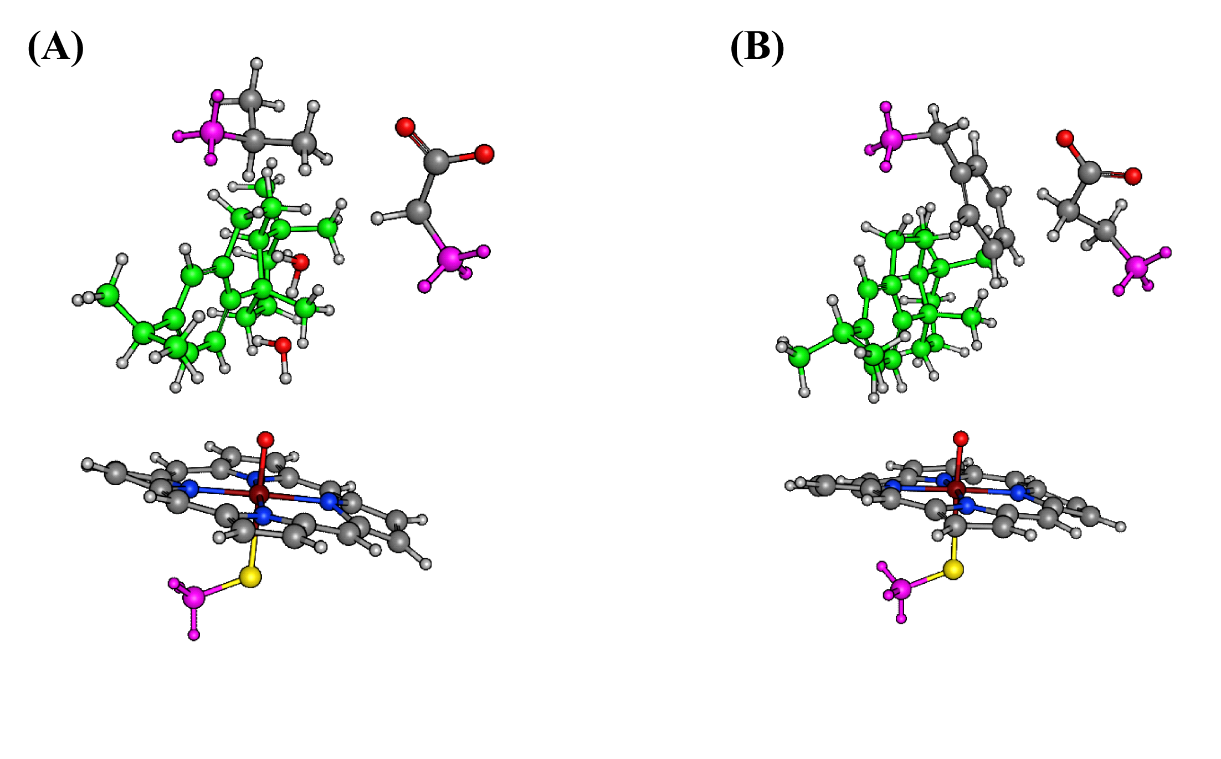


**Figure S4.** QM cluster models of complex (A) abi-wt (B) abi-mut. Magenta atoms are frozen. The wt-abi complex has two more water molecules. The wt-abi complex has two more water molecules as observed from the MD trajectory of system mut-abi-Cpd I.


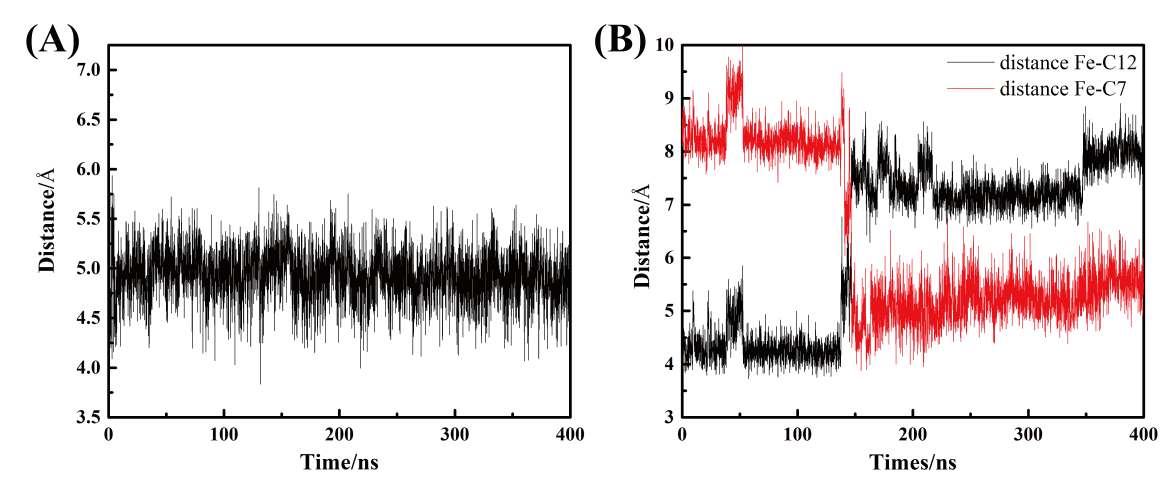


**Figure S5.** Distances evolution in systems (A) wt-abi-Rest (B) mut-abi-Rest.


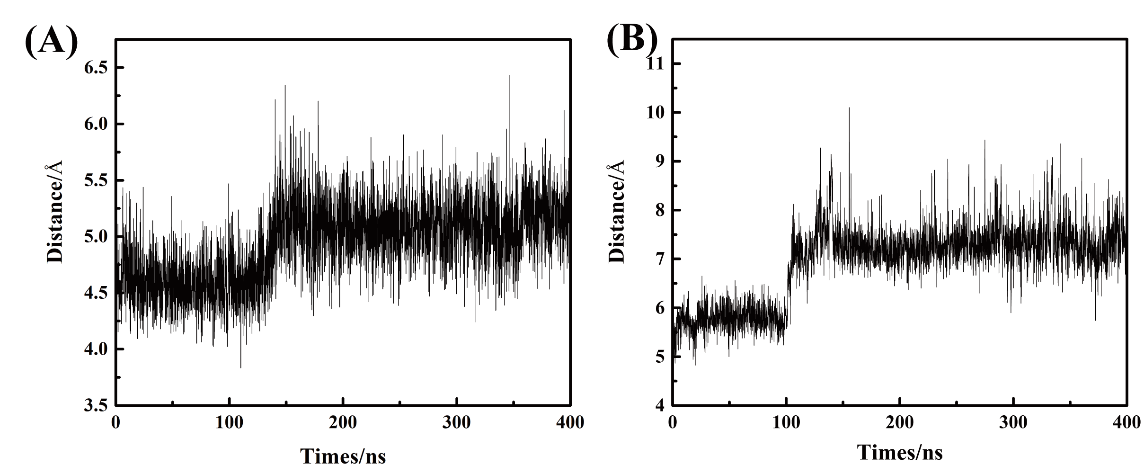


**Figure S6.** Distances evolution in systems (A) mut-fer-C7-Rest (B) mut-fer-C11-Rest.


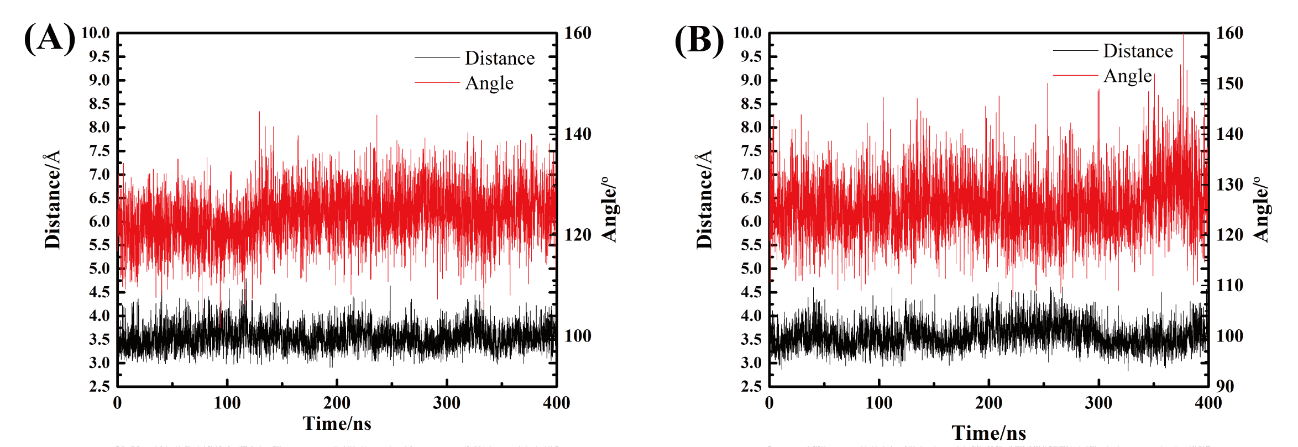


**Figure S7.** Evolution of distance and Fe-O-C12 angle in systems (A) wt-abi-Cpd I and (B) mut-abi-Cpd I


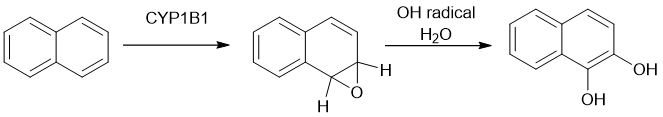


**Figure S8.** 1,2-naphalenediol can be transformed from epoxidation medium with OH radical and water molecule.

**Cartesian coordinates of reaction species for complex wt-abi**

**wt-abi**

S -5.71052300 0.03314700 0.06917400

C -6.29903300 -1.16543400 -1.15214400

H -6.50047500 -2.12934600 -0.68335200

H -5.58350000 -1.29563300 -1.96196800

H -7.22859100 -0.75568500 -1.55009300

C 7.47544300 -1.84156700 -0.78099900

C 7.02627000 -1.41843100 0.62756800

C 7.72896000 -2.24370400 1.70863200

C 7.21948400 0.08317100 0.84585800

H 7.18969600 -2.87998000 -0.94869000

H 6.94472200 -1.23561900 -1.51446200

H 8.52712300 -1.60127000 -0.94196400

H 5.94215300 -1.62718200 0.70198600

H 8.82367100 -2.08016400 1.68173000

H 7.37316900 -1.96983300 2.71918700

H 7.54669000 -3.32536200 1.56840700

H 6.84584000 0.39954400 1.83723200

H 8.28680200 0.36720000 0.78371500

H 6.68219400 0.67023400 0.08061800

C 5.21604000 5.05104700 0.86488700

C 6.30585000 3.97781500 0.89688300

C 7.19004300 3.90873700 -0.36541500

O 6.90163100 4.65884900 -1.33548500

O 8.14569100 3.08236300 -0.33919500

H 5.66584000 6.04023700 0.78602600

H 4.65213000 5.02508100 1.79770500

H 4.61306600 4.90119700 -0.03020000

H 6.97539900 4.11031400 1.76746300

H 5.84950500 2.97728300 1.02941600

C -3.35101400 -3.15837500 -0.32700400

C -2.68481200 0.56558200 -3.36043600

C -3.81042400 3.62266500 0.22869300

C -3.55970500 -0.04701800 3.38376300

C -3.13237800 -2.40375000 -1.47537300

C -2.83877900 -2.97155800 -2.76969500

C -2.60257500 -1.92377300 -3.61523000

C -2.77720600 -0.71737200 -2.84142400

C -2.94262200 1.72800000 -2.64146300

C -2.95718600 3.05319200 -3.21317200

C -3.32423400 3.90874000 -2.21112100

C -3.50593800 3.10527700 -1.02559100

C -3.81938100 2.88867700 1.40775100

C -3.96261900 3.46182600 2.72501300

C -3.82907900 2.43479200 3.61760300

C -3.63749700 1.23206000 2.84218900

C -3.53005200 -1.21654400 2.64159000

C -3.55368200 -2.54462100 3.20454100

C -3.53995900 -3.42263500 2.15714100

C -3.48446300 -2.63484600 0.94911400

N -3.10000300 -1.03355300 -1.54113000

N -3.27388400 1.78338000 -1.31195000

N -3.62827700 1.53226100 1.50381300

N -3.50147400 -1.29174900 1.26406400

H -2.80861700 -4.04051100 -2.97875100

H -2.34538400 -1.94291100 -4.67394000

H -2.72616500 3.27694000 -4.25432300

H -3.45404500 4.99007800 -2.24709900

H -4.12937500 4.52093000 2.92022700

H -3.87214000 2.46448700 4.70613000

H -3.57221600 -2.75686900 4.27295400

H -3.54646500 -4.51211500 2.17893000

H -3.34501200 -4.24499000 -0.42996800

H -2.42320700 0.66899400 -4.41516900

H -3.97738100 4.69890400 0.30305700

H -3.57085300 -0.13848300 4.47141000

Fe -3.22385500 0.25821700 -0.00662700

O -1.60191400 0.31200900 0.24793200

C 1.77176600 -4.31401000 -2.21206700

C 3.04315100 -4.69203800 -1.45633800

C 3.11473500 -4.12941000 -0.01876400

C 2.81662500 -2.59497600 -0.06451600

C 1.54775000 -2.14943600 -0.86292000

C 1.58999100 -2.79648500 -2.26589800

C 2.84947000 -1.90419500 1.30407000

C 3.00088700 -0.39516100 1.12746600

C 2.09333000 0.18924400 0.06460500

C 1.52648500 -0.61343500 -0.95088200

C 1.82872000 1.56686800 0.08528300

C 1.02023100 2.19234000 -0.87288300

C 0.54763600 1.40539600 -1.92953800

C 0.79357000 0.03131800 -1.96018900

C 4.55874800 -4.34642400 0.47590500

C 2.18460500 -4.92308800 0.91793400

C 0.20034400 -2.51140500 -0.18900400

C 0.57926200 3.63610400 -0.71151700

C 1.75562200 4.61793300 -0.66792100

C -0.30043300 3.77306500 0.54312000

H 0.89052800 -4.78428300 -1.74122800

H 1.81978300 -4.71346000 -3.24135500

H 3.91086100 -4.30078700 -2.02167400

H 3.15974200 -5.79139400 -1.42114500

H 0.66351100 -2.56351400 -2.81747500

H 2.42733100 -2.35328900 -2.83734400

H 1.93229200 -2.12334800 1.87392900

H 3.69125700 -2.27469800 1.90984600

H 4.04839500 -0.17620600 0.83846500

H 2.84032800 0.12802400 2.08703400

H 2.24632500 2.15981400 0.90651400

H -0.07421400 1.85995300 -2.70622300

H 0.34390300 -0.55450700 -2.76415400

H 4.68861400 -4.07088000 1.53633000

H 5.27487300 -3.75552000 -0.12044800

H 4.83503600 -5.41206600 0.37660300

H 2.12177300 -4.45773000 1.91694100

H 2.57883200 -5.94697600 1.05264100

H 1.15970000 -5.01930700 0.53023600

H 3.66236800 -2.17569600 -0.64476200

H 0.10215200 -2.06092800 0.80895800

H 0.04189200 -3.59487200 -0.09243500

H -0.61948600 -2.11143100 -0.80367000

H -0.04702000 3.88417700 -1.58698100

H 1.39325200 5.66002100 -0.59733600

H 2.38545200 4.53484600 -1.57169000

H 2.39656400 4.42688100 0.20985200

H 0.29233800 3.60606800 1.46090400

H -1.11425300 3.03218400 0.53651200

H -0.74868800 4.78146700 0.60404800

O -0.33362100 -1.99269100 3.32626300

H -0.91070400 -2.44091200 2.68406100

H -0.30348800 -1.04816900 3.01263300

O -0.26906200 0.61843100 2.52160900

H -0.82372900 0.53404300 1.69540600

H 0.62051000 0.81401400 2.17509000

**wt-TS1**

S 5.64560500 -0.05135400 0.23785700

C 6.32374400 0.97323500 -1.12153500

H 6.46663700 2.00497700 -0.79817400

H 5.66138900 0.94954900 -1.98494500

H 7.28893500 0.54195800 -1.39124700

C -7.45764500 1.18172300 -1.72417300

C -7.10641400 0.99315700 -0.23910800

C -7.97759100 1.87847100 0.65619600

C -7.19414700 -0.47803100 0.17213300

H -7.18950600 2.19155200 -2.03459000

H -6.86206800 0.48980400 -2.31877200

H -8.48897600 0.88003000 -1.91097500

H -6.05667800 1.31332800 -0.10628600

H -9.04095000 1.57694900 0.59114800

H -7.67101600 1.80414200 1.71599100

H -7.91111500 2.94108300 0.35964200

H -6.89891600 -0.62045200 1.22816700

H -8.22121700 -0.87091300 0.05609400

H -6.53322700 -1.10788000 -0.44943500

C -5.12770200 -5.28309900 1.12134200

C -6.25170600 -4.26447600 0.91797700

C -7.07942100 -4.45122400 -0.37131200

O -6.74305700 -5.36727500 -1.16810900

O -8.04750100 -3.65642500 -0.54020000

H -5.54390500 -6.28900100 1.17071500

H -4.62910000 -5.09222600 2.07199600

H -4.46976600 -5.25155300 0.25335900

H -6.95817100 -4.27645900 1.76933500

H -5.83803900 -3.23747900 0.89717800

C 3.05338300 3.09963800 0.86737500

C 3.24894200 0.63574800 -3.31010500

C 3.88729200 -3.47434300 -0.81016300

C 3.24241600 -1.05880500 3.34287600

C 3.11172700 2.77889000 -0.48194800

C 2.98621500 3.75003100 -1.54222500

C 2.99893300 3.05539000 -2.71963300

C 3.15061800 1.66127800 -2.37905000

C 3.45410800 -0.69891200 -2.98617900

C 3.61656200 -1.75880700 -3.95306100

C 3.80855900 -2.91215900 -3.24558800

C 3.75555200 -2.55956000 -1.84589300

C 3.79126600 -3.16759800 0.54161300

C 3.84806900 -4.14524100 1.60460100

C 3.64153600 -3.46704100 2.77262300

C 3.47257700 -2.07421900 2.42249600

C 3.10637400 0.28544200 3.02376100

C 2.92359600 1.34020700 3.99664800

C 2.89260100 2.51595200 3.30102900

C 3.04414500 2.17499500 1.90317200

N 3.21686500 1.51367800 -1.01369700

N 3.53693200 -1.20805600 -1.71215400

N 3.57677300 -1.91960500 1.06376500

N 3.16387700 0.81836100 1.76140600

H 2.88594700 4.82326500 -1.38169500

H 2.91903600 3.43342200 -3.73858800

H 3.58842200 -1.61446300 -5.03286200

H 3.97077000 -3.92368200 -3.61748600

H 4.00976600 -5.21203200 1.45123300

H 3.60250600 -3.85339900 3.79099200

H 2.83292900 1.17731300 5.07016100

H 2.77656400 3.53217500 3.67748200

H 2.95343200 4.15453300 1.12940100

H 3.18749800 0.90009300 -4.36727800

H 4.05159000 -4.51937900 -1.07992200

H 3.17628300 -1.33769700 4.39628800

Fe 3.31165300 -0.20675700 0.00434000

O 1.59325900 -0.44300000 0.13122200

C -1.64669200 4.76638900 -1.58424500

C -3.12165000 4.90546700 -1.21471700

C -3.52848000 4.14256600 0.06651100

C -3.01728100 2.67016700 -0.04381900

C -1.52287900 2.47614800 -0.45521900

C -1.25119800 3.29720400 -1.73688700

C -3.34523200 1.78287100 1.16309100

C -3.25591500 0.31165700 0.76260500

C -2.03865500 -0.02466600 -0.05865900

C -1.26350400 0.98135500 -0.69139000

C -1.67658900 -1.38709900 -0.18461500

C -0.52549000 -1.79186200 -0.84237200

C 0.28366000 -0.77407700 -1.43086200

C -0.14879800 0.57670100 -1.41715700

C -5.06895100 4.13407500 0.11659800

C -3.03337100 4.88784700 1.31961000

C -0.48798100 2.87156800 0.62631800

C -0.06581800 -3.23468800 -0.86289300

C -1.11048400 -4.16415000 -1.49799000

C 0.30805400 -3.70995100 0.55193600

H -1.01177800 5.25151700 -0.82223300

H -1.45036500 5.29827500 -2.53271200

H -3.72674500 4.51338500 -2.05471700

H -3.39289900 5.97165000 -1.10158000

H -0.18448000 3.23561600 -2.01030200

H -1.82623000 2.85354200 -2.57141800

H -2.65123500 1.98475300 1.99588300

H -4.35944700 1.98419200 1.54046800

H -4.15082600 0.04277900 0.16592400

H -3.28407500 -0.34481200 1.65033500

H -2.31588200 -2.13198700 0.30001100

H 1.09109600 -1.06897000 -2.09988500

H 0.48096700 1.30935900 -1.92160000

H -5.45626100 3.70673600 1.05664600

H -5.49216300 3.55331300 -0.72181300

H -5.45471700 5.16710900 0.04090600

H -3.20287700 4.29490700 2.23523700

H -3.58916700 5.83677000 1.43106500

H -1.96379000 5.14162100 1.27774500

H -3.58859600 2.25427300 -0.89707200

H -0.59784800 2.27973600 1.54652100

H -0.52909600 3.93665700 0.89428700

H 0.52116900 2.66563800 0.23802200

H 0.84843800 -3.27375000 -1.47998500

H -0.72148300 -5.19692900 -1.55266200

H -1.36775100 -3.83988900 -2.52195000

H -2.04169500 -4.18661700 -0.90209000

H -0.58655100 -3.75575700 1.19934800

H 1.03101300 -3.02586400 1.02071200

H 0.75554900 -4.71896100 0.51325700

O -0.38747200 1.45439200 3.83195800

H 0.34464600 2.05698800 3.61074700

H -0.21325100 0.66238700 3.25235600

O 0.11741000 -0.73877200 2.33379000

H 0.69176400 -0.56630000 1.52989000

H -0.69739200 -1.11256000 1.95237300

**wt-IM1**

S -5.21913400 -0.90461300 -1.66283900

C -6.04098600 0.45594200 -2.57838300

H -6.10614700 0.18786300 -3.63354600

H -5.51971200 1.40583800 -2.47450600

H -7.04677300 0.54559400 -2.16534100

C 7.48555900 3.15031100 -2.25202500

C 7.41482500 1.61901700 -2.15920900

C 8.35079000 0.95508900 -3.17331300

C 7.72373200 1.15455000 -0.73371800

H 7.19887000 3.48661000 -3.24838300

H 6.78841100 3.59791800 -1.54452000

H 8.46803100 3.46940700 -1.90196500

H 6.38134800 1.31095600 -2.40617500

H 9.40453600 1.23080000 -2.97556500

H 8.27719700 -0.14699300 -3.12549100

H 8.11063900 1.26523100 -4.20715800

H 7.62199700 0.05946200 -0.62839000

H 8.75760700 1.42461900 -0.44682600

H 7.04846300 1.62737900 -0.00105600

C 5.45570400 -0.46130800 3.92495500

C 6.57103500 -0.03487400 2.96807800

C 7.13329800 1.38217300 3.19456900

O 6.51940500 2.14834700 3.98452100

O 8.17324000 1.68585200 2.54360900

H 5.84876700 -0.51756900 4.93976000

H 5.11389700 -1.46072700 3.65451800

H 4.67340500 0.29714600 3.92664300

H 7.41761600 -0.74479300 3.00306100

H 6.20826700 -0.06862800 1.92392700

C -1.45999000 -1.63919200 -2.57402800

C -3.23468900 2.84977500 -2.06623800

C -5.71163900 1.37371200 1.83296000

C -3.73436900 -3.03760900 1.47547500

C -1.74230200 -0.29585500 -2.78567500

C -1.23179500 0.48289300 -3.88936000

C -1.71789600 1.75315900 -3.73921000

C -2.53148400 1.75187500 -2.54656000

C -4.07702800 2.82840900 -0.95882200

C -4.80025000 3.98137600 -0.46610700

C -5.50904400 3.56092300 0.62337900

C -5.21020900 2.15354700 0.79548100

C -5.38409400 0.04229100 2.07310800

C -5.88005500 -0.72545400 3.19726900

C -5.30772400 -1.96261100 3.10910900

C -4.47168100 -1.94872100 1.92710900

C -2.95521100 -3.04375000 0.32378900

C -2.21250700 -4.19227600 -0.15543900

C -1.57940300 -3.79714200 -1.30063700

C -1.93470400 -2.40983700 -1.51476100

N -2.51511400 0.50232700 -1.96509700

N -4.34368700 1.73120200 -0.17976000

N -4.53590400 -0.72283800 1.31745900

N -2.76693400 -1.97681300 -0.51608400

H -0.58322400 0.09013400 -4.67254800

H -1.55544000 2.62414800 -4.37414800

H -4.75424900 4.97539800 -0.91086900

H -6.17271300 4.13481600 1.27036600

H -6.57103200 -0.34368000 3.94912900

H -5.42717000 -2.82041700 3.77114200

H -2.19226200 -5.16511200 0.33586300

H -0.91952700 -4.37311300 -1.94929700

H -0.80495800 -2.13200700 -3.29554100

H -3.13401200 3.79236700 -2.60796000

H -6.39839600 1.85432600 2.53334100

H -3.77433600 -3.95640600 2.06383800

Fe -3.58607000 -0.13211000 -0.36247400

O -2.09329200 0.40371800 0.90672900

C 2.64531400 -3.87213400 -0.75718000

C 3.72542900 -3.45543000 -1.75157700

C 4.55429300 -2.23350300 -1.29745200

C 3.57659600 -1.08439700 -0.88836800

C 2.40188500 -1.46272500 0.06862200

C 1.68662900 -2.71666500 -0.47796400

C 4.27637900 0.17461100 -0.37409300

C 3.32957800 1.37095000 -0.37128500

C 1.91797300 1.07599700 0.04309300

C 1.43884300 -0.26772700 0.11983700

C 1.06528600 2.16913500 0.29783500

C -0.28290700 2.01214500 0.56863200

C -0.86005500 0.66524400 0.41049800

C 0.09360300 -0.44490500 0.33661100

C 5.38696700 -1.76759500 -2.50815300

C 5.54649200 -2.64102300 -0.19169600

C 2.81550300 -1.70584600 1.54015400

C -1.20643900 3.11729600 0.98116100

C -0.87339100 4.48785500 0.39476200

C -1.24489700 3.13568600 2.52535600

H 3.10257300 -4.22534100 0.18381700

H 2.07251500 -4.72568900 -1.16276100

H 3.23457100 -3.20405700 -2.71156700

H 4.41037000 -4.29866700 -1.95751700

H 0.91250600 -3.04841500 0.23049700

H 1.16856400 -2.44852000 -1.41609900

H 4.66777000 0.00551500 0.63942200

H 5.13943500 0.42239500 -1.00297600

H 3.25397900 1.77444300 -1.40170500

H 3.72444800 2.20407100 0.23662200

H 1.50512500 3.16953600 0.29779500

H -0.97111300 0.79949400 -0.74010000

H -0.34739500 -1.43550400 0.41214400

H 6.12405500 -0.99567700 -2.23498800

H 4.74129900 -1.35770200 -3.30605800

H 5.94687700 -2.62095400 -2.93210700

H 6.08292900 -1.76693900 0.21582900

H 6.30358900 -3.32813000 -0.61151400

H 5.06688400 -3.16212800 0.65049800

H 3.07495700 -0.80758100 -1.83655200

H 3.27069800 -0.81107300 1.99264000

H 3.52587500 -2.53782700 1.64680900

H 1.91482900 -1.95469100 2.12703600

H -2.20963800 2.82029900 0.64836300

H -1.66325100 5.20939200 0.66816100

H -0.81818700 4.44486200 -0.70724200

H 0.08630800 4.88027100 0.77545600

H -0.26679900 3.43948200 2.93923500

H -1.50116400 2.14124900 2.92757400

H -2.00924700 3.85562400 2.86811100

O -0.56263000 -3.10286300 2.58022500

H -0.86469700 -3.29402200 1.67538500

H -0.93965600 -2.20109200 2.78027700

O -1.54379300 -0.63879600 3.20068500

H -1.85221800 -0.26089000 2.30637800

H -0.71969300 -0.15112600 3.38126400

**Cartesian coordinates of reaction species for complex mut-abi**

**mut-abi**

C 7.52161400 1.69238900 0.83376000

C 7.48929500 0.63020900 -0.28540800

C 6.08573900 0.21722700 -0.66631100

C 5.41738600 0.80407100 -1.75096900

C 4.09618000 0.45580900 -2.05575700

C 3.41897000 -0.48796700 -1.27756900

C 4.07693900 -1.08906000 -0.19764500

C 5.39502400 -0.73613500 0.10319100

H 7.02360500 2.60321100 0.50221200

H 6.87139400 1.36275000 1.64363700

H 8.52411700 1.72765200 1.26057700

H 8.01878200 1.01512300 -1.17497900

H 8.04394200 -0.26174500 0.05705700

H 5.93725600 1.54889700 -2.36212400

H 5.90073900 -1.20521900 0.95263400

H 3.59127100 0.93029600 -2.90216200

H 3.55681500 -1.83125200 0.41543300

H 2.38228900 -0.75109900 -1.50684900

C 4.66324200 -5.65757100 -1.31179800

C 5.53755300 -4.41601000 -1.53921000

C 6.49116500 -4.11522500 -0.38439500

C 7.60363400 -5.15829700 -0.14135000

O 7.94501500 -5.89773300 -1.10573900

O 8.12437200 -5.16995000 1.00726400

H 5.29723600 -6.53636100 -1.19270700

H 4.11980000 -5.55466400 -0.37280500

H 3.96966800 -5.76911500 -2.14515300

H 6.13029600 -4.54957700 -2.46245700

H 4.88668900 -3.53662400 -1.70459800

H 7.00206300 -3.14934600 -0.56752400

H 5.93171400 -3.97906100 0.55930000

S -5.98224500 -1.18298500 -0.18569800

C -6.70826400 0.17328400 0.76590700

H -7.78975500 0.15967200 0.62716000

H -6.30997600 1.12950700 0.42759100

H -6.47534700 0.03495800 1.82165600

C -3.50199700 2.39725700 -1.60855500

C -3.89372800 0.42570000 2.80384300

C -3.42334900 -3.98641100 0.85349100

C -3.80405100 -2.02864000 -3.56326500

C -3.60553600 2.24127300 -0.23373200

C -3.68440700 3.33510200 0.70634500

C -3.82194200 2.77787100 1.94638000

C -3.81181700 1.34553400 1.76257200

C -3.80897100 -0.95057400 2.65550400

C -3.77384600 -1.89221000 3.74913000

C -3.59516300 -3.13081700 3.20081400

C -3.54760600 -2.95407000 1.76953600

C -3.48926800 -3.83356400 -0.52833000

C -3.50764400 -4.93076900 -1.46807900

C -3.66372900 -4.38220400 -2.70994700

C -3.71360900 -2.94991700 -2.52753400

C -3.73500700 -0.64731000 -3.41090700

C -3.69955100 0.28844700 -4.51051900

C -3.56538900 1.53343000 -3.96181300

C -3.54483900 1.35419400 -2.52900600

N -3.68937600 1.03890600 0.43110500

N -3.68770100 -1.61660800 1.45515600

N -3.61235200 -2.64085300 -1.19351000

N -3.64780600 0.02293000 -2.21734000

H -3.64358600 4.38803200 0.42970000

H -3.91564400 3.27260300 2.91261200

H -3.85888500 -1.61600200 4.79959500

H -3.50801300 -4.09371300 3.70344400

H -3.42631900 -5.98123300 -1.18942300

H -3.73139100 -4.88276800 -3.67564600

H -3.76086800 0.00649600 -5.56143400

H -3.49988600 2.49901300 -4.46284400

H -3.42862600 3.41460100 -1.99788100

H -3.98254300 0.81913700 3.81807200

H -3.31777000 -4.99992800 1.24538500

H -3.88154600 -2.41911000 -4.58004300

Fe -3.48929000 -0.79098600 -0.38762400

O -1.85305400 -0.79189600 -0.38212000

C -0.03581000 5.73529700 -0.53788000

C 1.24349700 6.49454100 -0.19340900

C 2.53751900 5.67685700 -0.40648900

C 2.38169500 4.30084800 0.31556300

C 1.07484500 3.49330900 0.02176900

C -0.13752200 4.42710900 0.24755200

C 3.60396000 3.38264000 0.20860000

C 3.55997300 2.31815900 1.30287500

C 2.19373100 1.69655600 1.49726400

C 1.02524700 2.28468600 0.97244400

C 2.09471500 0.50932900 2.24637000

C 0.87220900 -0.11519000 2.50803500

C -0.28765300 0.50195900 2.01569300

C -0.20522300 1.66735100 1.25906500

C 3.68647900 6.46176600 0.25784500

C 2.86813400 5.56608600 -1.90628300

C 0.98071000 2.89562900 -1.40432500

C 0.78740000 -1.43979600 3.24645500

C 0.30815500 -2.55413200 2.29991200

C -0.11035200 -1.34908000 4.48867500

H -0.07890900 5.53076900 -1.62207400

H -0.91454100 6.36583300 -0.31002800

H 1.19523400 6.79002900 0.87246800

H 1.30588500 7.43146100 -0.77796300

H -1.06979300 3.91014700 -0.03033400

H -0.21089800 4.65922100 1.32672600

H 3.63695300 2.90157000 -0.78105200

H 4.54136500 3.95275300 0.30716700

H 3.86311600 2.78426500 2.26140000

H 4.30112800 1.52619700 1.10498900

H 3.01718000 0.05372100 2.62281100

H -1.26502500 0.05284500 2.19422300

H -1.12671300 2.09019400 0.85962200

H 3.68868700 7.50693100 -0.10190900

H 4.67635600 6.03346100 0.02456100

H 3.57417200 6.48330300 1.35723300

H 3.15565900 6.55884600 -2.29851800

H 2.02142800 5.21069200 -2.51184600

H 3.71676300 4.88203200 -2.08183300

H 2.28983400 4.57302200 1.38562100

H 1.84679900 2.25680200 -1.64031100

H 0.89915100 3.66451900 -2.18608600

H 0.07836700 2.26190200 -1.46965700

H 1.80732200 -1.69864700 3.58218900

H 0.99323800 -2.66195800 1.43926100

H -0.69863900 -2.33505800 1.90130100

H 0.26178300 -3.52527900 2.82595600

H 0.24411600 -0.56458400 5.18120000

H -0.11969400 -2.30997700 5.03484900

H -1.15280900 -1.11453700 4.21023800

**mut-TS1**

C 7.32384600 2.99087100 0.83546300

C 7.52430800 1.83259700 -0.16203600

C 6.22337000 1.33231200 -0.75362300

C 5.55796300 2.06583900 -1.75177300

C 4.34646700 1.61882200 -2.28910700

C 3.77354100 0.42400300 -1.83793500

C 4.42071000 -0.31312800 -0.84135900

C 5.63022600 0.14170100 -0.30239500

H 6.75511900 3.78112900 0.34754600

H 6.63537000 2.66627200 1.61554400

H 8.26081700 3.21126300 1.34839700

H 8.18940000 2.17093400 -0.97787600

H 8.03822800 0.99625000 0.34303600

H 6.00101100 2.99797400 -2.11776400

H 6.12520500 -0.44038100 0.47978800

H 3.84665200 2.20651900 -3.06493700

H 3.97571500 -1.24162300 -0.47180500

H 2.82319700 0.07566700 -2.25276100

C 5.67029600 -4.87703300 -0.63491300

C 6.43091900 -3.57872600 -0.93565800

C 7.29220500 -3.09306100 0.22953000

C 8.47178800 -4.01387400 0.61377600

O 8.94686600 -4.77111100 -0.27778300

O 8.90776500 -3.91425200 1.79275300

H 6.39248000 -5.64074700 -0.34684700

H 5.01717900 -4.74609900 0.22789900

H 5.09262400 -5.17529200 -1.50976400

H 7.08703700 -3.73967100 -1.81031100

H 5.71630600 -2.78491400 -1.21826700

H 7.73513700 -2.10932400 -0.02212500

H 6.67579600 -2.92262100 1.13111700

S -5.40250600 -1.76559000 -1.08496200

C -6.50454800 -0.47922000 -0.39330300

H -7.55406100 -0.65810800 -0.63149000

H -6.19552000 0.47892700 -0.80918800

H -6.37521700 -0.46603000 0.68879800

C -4.38236700 1.75272500 1.13830900

C -3.89058100 -2.87884100 2.51416400

C -2.03134200 -3.97976700 -1.82272700

C -2.95784500 0.53347800 -3.33335500

C -4.33113900 0.59599600 1.90393700

C -4.75607400 0.51906700 3.28061400

C -4.65009000 -0.79088300 3.65583600

C -4.14440800 -1.51497000 2.51449600

C -3.34840400 -3.58349000 1.44614700

C -3.00238900 -4.98693300 1.48564600

C -2.44845900 -5.28778900 0.27368500

C -2.47208500 -4.07139700 -0.50867100

C -2.11582100 -2.83746400 -2.60964400

C -1.71533500 -2.77205100 -3.99812000

C -1.99917200 -1.50748800 -4.43020600

C -2.56155600 -0.79837500 -3.30173600

C -3.44196300 1.24695700 -2.24532000

C -3.80503400 2.64565400 -2.29312700

C -4.18737300 2.99847000 -1.03009200

C -4.06691000 1.81246700 -0.21311500

N -3.94233000 -0.64879600 1.46083000

N -3.01762700 -3.05189700 0.22927100

N -2.62452000 -1.62774700 -2.21277400

N -3.61416400 0.76217900 -0.97048300

H -5.10359600 1.37177400 3.86353600

H -4.88661700 -1.24852600 4.61626000

H -3.16254700 -5.63644600 2.34600100

H -2.05916300 -6.24099900 -0.08366100

H -1.28167300 -3.60394700 -4.55304800

H -1.84617300 -1.07067700 -5.41702800

H -3.75382900 3.26293200 -3.18974700

H -4.52084500 3.96807500 -0.66129900

H -4.72070300 2.66941100 1.62410000

H -4.10097500 -3.43111100 3.43194900

H -1.61052600 -4.87811200 -2.27870900

H -2.85989700 1.06646800 -4.28121700

Fe -3.22421600 -1.11511100 -0.33812300

O -1.54175700 -0.79809800 -0.04022800

C -1.39188500 5.58790800 0.54876200

C -0.14171200 6.40651300 0.86351300

C 1.16113300 5.81772800 0.27647600

C 1.25253000 4.31129000 0.67939000

C -0.01233700 3.43551000 0.39866500

C -1.24184100 4.13985200 1.01538000

C 2.51757300 3.59723600 0.19021600

C 2.73286700 2.31125700 0.98196500

C 1.49177400 1.47550400 1.14293500

C 0.19786200 2.04398700 1.01638700

C 1.62702600 0.11788200 1.51350900

C 0.54374900 -0.70174300 1.79499600

C -0.76358000 -0.12100600 1.71214600

C -0.88921400 1.25413900 1.36817100

C 2.33706400 6.57939400 0.92006500

C 1.23011500 6.06702200 -1.24133900

C -0.28654100 3.15086000 -1.10085800

C 0.76772300 -2.15842200 2.15406900

C 0.76791000 -3.03398400 0.88667400

C -0.22980800 -2.68845200 3.18871700

H -1.60755200 5.61596300 -0.53372000

H -2.26785800 6.04120200 1.04709700

H -0.02993300 6.45998000 1.96360900

H -0.26133200 7.44669400 0.50740700

H -2.16024700 3.58575600 0.76928500

H -1.14077700 4.12407700 2.11684700

H 2.44604000 3.36525000 -0.88484700

H 3.40588400 4.23602000 0.31144300

H 3.07060700 2.57967500 2.00429100

H 3.54332700 1.70370900 0.55226300

H 2.63822600 -0.29174200 1.59999900

H -1.59595100 -0.60342100 2.22223900

H -1.89465700 1.66480600 1.33400600

H 2.18456900 7.66898500 0.81433700

H 3.30335100 6.33683100 0.44544100

H 2.41960700 6.35298900 1.99878800

H 1.35056900 7.14868900 -1.43484600

H 0.32538000 5.73999100 -1.77429700

H 2.09371500 5.55117300 -1.69607100

H 1.32026700 4.33519000 1.78478400

H 0.60342700 2.74561500 -1.60957100

H -0.61837800 4.04271100 -1.65106100

H -1.08344200 2.39374300 -1.18623400

H 1.77966600 -2.22135000 2.59172300

H 1.53723900 -2.69162000 0.17130500

H -0.21002700 -2.99511000 0.38181800

H 0.98305100 -4.08629400 1.14695400

H -0.25292400 -2.05193200 4.09134200

H 0.05093600 -3.71085200 3.49769800

H -1.25038700 -2.73551600 2.77721300

**mut-IM1**

C -7.49606100 1.96106900 -2.14160400

C -7.62714900 1.13385900 -0.84663800

C -6.30591400 0.70890600 -0.24571100

C -5.77303400 1.34364600 0.88599900

C -4.55510800 0.92798300 1.43876300

C -3.84344400 -0.12798900 0.86268100

C -4.36265100 -0.76978600 -0.26827100

C -5.57790600 -0.35231100 -0.81567000

H -6.98196600 2.90045000 -1.94373400

H -6.79941700 1.45978400 -2.81340700

H -8.45718000 1.94735900 -2.65713900

H -8.20310000 1.70819200 -0.09971700

H -8.21641100 0.22613900 -1.07103500

H -6.32484700 2.16866700 1.34784500

H -5.98165800 -0.86744500 -1.69300400

H -4.16464300 1.43133500 2.32752700

H -3.81808000 -1.60268500 -0.72187000

H -2.88792400 -0.44862600 1.28735700

C -5.20146600 -4.93603500 1.59533300

C -6.02584200 -3.64426800 1.50307300

C -6.88694200 -3.55885200 0.24357400

C -8.01882800 -4.60580800 0.14937900

O -8.51380700 -5.03678200 1.22809000

O -8.39699800 -4.93301500 -1.00805300

H -5.86686000 -5.79918300 1.58593300

H -4.57741100 -5.03305700 0.70697100

H -4.58548600 -4.90936700 2.49416900

H -6.68662600 -3.56724500 2.38537300

H -5.34631700 -2.77254400 1.53751300

H -7.37071500 -2.56307900 0.19293600

H -6.26237800 -3.63592700 -0.66491200

S 5.70035100 -1.10122400 0.66896700

C 6.58323200 -0.00020300 -0.49815900

H 7.64796300 -0.02418000 -0.26143400

H 6.20796300 1.01585900 -0.38284300

H 6.43250900 -0.33041700 -1.52579800

C 3.83247500 0.92156900 -2.65778900

C 3.95969100 -3.79723300 -1.54372900

C 2.59652000 -2.74522400 2.98792500

C 3.55507300 1.91931300 2.07782300

C 3.89768000 -0.46623700 -2.76813400

C 4.15381100 -1.18001200 -4.00151600

C 4.26702200 -2.50223700 -3.67124000

C 4.04535600 -2.59639500 -2.24351500

C 3.58094200 -3.92514900 -0.20949100

C 3.33853000 -5.19132800 0.45426700

C 2.90424700 -4.89576200 1.71589800

C 2.90785300 -3.45031600 1.82706000

C 2.73020400 -1.36746900 3.13629200

C 2.55210300 -0.65459800 4.38523100

C 2.87154600 0.65156600 4.13931900

C 3.21860300 0.73740300 2.73454600

C 3.74429000 2.04822200 0.70426600

C 3.94907000 3.31103800 0.02544100

C 3.94714200 3.04309100 -1.31463000

C 3.78025400 1.61099100 -1.45036300

N 3.82757700 -1.34709500 -1.72158700

N 3.31806900 -2.89004500 0.64755900

N 3.12238300 -0.49811700 2.15252200

N 3.65059200 1.03246300 -0.21351300

H 4.25151300 -0.70977800 -4.98016200

H 4.46417000 -3.35316100 -4.32367200

H 3.47453000 -6.16905900 -0.00877400

H 2.61468800 -5.57767900 2.51579700

H 2.23851900 -1.11593000 5.32200700

H 2.86924700 1.49622100 4.82875800

H 4.04600400 4.27355900 0.52795700

H 4.06255700 3.73480200 -2.14919400

H 3.91716700 1.50811200 -3.57504400

H 4.14050500 -4.71695900 -2.10474200

H 2.28071000 -3.32052200 3.86114200

H 3.61746000 2.82865400 2.67956100

Fe 3.48879900 -0.92496300 0.21338700

O 1.50712600 -0.99014900 -0.06645200

C 0.56950900 5.49244100 1.15505500

C -0.31940900 6.36374000 0.27117700

C -1.74702000 5.80578500 0.07517700

C -1.64159000 4.31585600 -0.38141300

C -0.71263200 3.38513400 0.46464900

C 0.66415900 4.06720500 0.61233900

C -2.98831700 3.63031100 -0.63071900

C -2.79055800 2.38903000 -1.49464100

C -1.59374000 1.55842800 -1.14414900

C -0.56856700 2.04518800 -0.27973500

C -1.50175900 0.27468500 -1.71682100

C -0.46550500 -0.60273800 -1.46434900

C 0.71053300 -0.10384300 -0.69096300

C 0.50364900 1.21505700 -0.04631200

C -2.40744600 6.62343200 -1.05279000

C -2.58853400 6.01694700 1.34741100

C -1.25555100 3.01997000 1.86589600

C -0.53639500 -2.01225900 -1.98533600

C -0.78644200 -2.98818700 -0.81719300

C 0.69316100 -2.41257800 -2.80982700

H 0.18828400 5.48081800 2.19120200

H 1.58492600 5.92570300 1.20507200

H 0.15728500 6.44926000 -0.72423000

H -0.38537400 7.38914600 0.67971500

H 1.31383500 3.47040000 1.27050100

H 1.15381400 4.09167100 -0.37745300

H -3.46649800 3.35169500 0.32125600

H -3.68846600 4.30374200 -1.14709700

H -2.64619800 2.69761000 -2.55007000

H -3.68517900 1.74178400 -1.49896300

H -2.33235300 -0.06204400 -2.34230100

H 1.20613700 0.34061900 -1.64499800

H 1.30261800 1.51657100 0.62864400

H -2.31751300 7.70369000 -0.83770400

H -3.48285600 6.39927400 -1.15837000

H -1.92031700 6.43082000 -2.02601500

H -2.78694100 7.09549100 1.48515800

H -2.08955700 5.66063400 2.26084900

H -3.56489100 5.50691500 1.27566600

H -1.13490400 4.37560700 -1.36457700

H -2.24020700 2.53084800 1.80972500

H -1.34401900 3.89453100 2.52511300

H -0.56143100 2.30840800 2.34679800

H -1.41951900 -2.05646800 -2.64461900

H -1.70251400 -2.71583800 -0.26324200

H 0.05983200 -2.98964300 -0.11269600

H -0.91796300 -4.01047200 -1.21466500

H 0.85157300 -1.71729200 -3.65260700

H 0.55116400 -3.42705600 -3.22174200

H 1.59434500 -2.41124200 -2.18501700

**mut-TS2**

C -7.30989000 3.21391500 -0.61565200

C -7.50252200 2.02189600 0.34249200

C -6.19989300 1.52839600 0.93843000

C -5.50520300 2.30253000 1.88542000

C -4.29671400 1.86037600 2.43267600

C -3.75628600 0.62803900 2.04607300

C -4.43286100 -0.15022900 1.10225900

C -5.63829600 0.30125000 0.55053700

H -6.72786400 3.97627400 -0.10070000

H -6.63314200 2.91644800 -1.41601200

H -8.25004900 3.46503100 -1.10757000

H -8.18209300 2.32595000 1.16045600

H -7.99711200 1.19074700 -0.18948800

H -5.92232000 3.26348300 2.20418800

H -6.15301100 -0.31123000 -0.19397600

H -3.77397100 2.48086800 3.16680300

H -4.01301200 -1.10775000 0.78050800

H -2.80832300 0.28302100 2.46915100

C -5.72175200 -4.72613200 0.49533000

C -6.47401400 -3.43806700 0.85454400

C -7.27619700 -2.86134900 -0.31278000

C -8.39900400 -3.77299500 -0.85351000

O -9.00140600 -4.51381800 -0.02716000

O -8.66218000 -3.68465600 -2.08327700

H -6.45559500 -5.46946200 0.18333300

H -5.07691100 -4.56463900 -0.36805200

H -5.13768100 -5.06740800 1.35018500

H -7.16909100 -3.64817900 1.68785400

H -5.76307200 -2.67549800 1.22002100

H -7.76394100 -1.91956000 0.00685000

H -6.60872100 -2.59259600 -1.15110000

S 5.37081700 -1.73409400 0.95533600

C 6.49398300 -0.44278200 0.30529800

H 7.54440600 -0.64232000 0.52385600

H 6.19980600 0.49953900 0.76529400

H 6.35373100 -0.38130500 -0.77393100

C 4.41751600 1.72804400 -1.28862800

C 3.65677000 -2.90338100 -2.50986400

C 2.06056000 -3.87178700 1.96341500

C 2.98495100 0.71467900 3.23033400

C 4.28304100 0.55781300 -2.02676900

C 4.63360300 0.43122900 -3.42382400

C 4.43727600 -0.87854300 -3.75990400

C 3.95891800 -1.54792700 -2.56971500

C 3.17626600 -3.57057200 -1.38795000

C 2.84953700 -4.98076700 -1.36165400

C 2.39079000 -5.25375200 -0.10422400

C 2.44325000 -4.01089300 0.63445700

C 2.14054600 -2.68813700 2.68858000

C 1.76836600 -2.55804400 4.08125300

C 2.03788900 -1.26742800 4.43851700

C 2.57124200 -0.61327500 3.26165900

C 3.49077500 1.37378100 2.11543000

C 3.91205500 2.75910000 2.11608800

C 4.31102900 3.04954900 0.84252600

C 4.13404100 1.84049900 0.06778300

N 3.85623100 -0.65211800 -1.53190700

N 2.91425900 -3.00201300 -0.16803300

N 2.61449800 -1.49278500 2.21039800

N 3.63489600 0.83620400 0.86039900

H 4.99326100 1.25069800 -4.04613600

H 4.59787500 -1.36880400 -4.72025000

H 2.95787900 -5.65510900 -2.21121200

H 2.04257100 -6.20159200 0.30622500

H 1.36009200 -3.36541500 4.68947500

H 1.89790800 -0.78183000 5.40451900

H 3.88707700 3.41078100 2.98945400

H 4.68199100 3.99179500 0.43966800

H 4.78631500 2.61581200 -1.80584800

H 3.79866100 -3.48955000 -3.42033200

H 1.68145200 -4.75766900 2.47736100

H 2.90564100 1.28689000 4.15732100

Fe 3.29182700 -1.08590900 0.35009200

O 1.27660900 -0.65199800 -0.03704300

C 1.55572500 5.29754700 -0.79438800

C 0.33672500 6.14203100 -1.16024100

C -0.98326100 5.65600300 -0.51898700

C -1.13453900 4.12804900 -0.79997800

C 0.09066500 3.23075500 -0.42995000

C 1.33562800 3.82186500 -1.12761000

C -2.43287600 3.50530900 -0.27957600

C -2.67969300 2.16881000 -0.96904600

C -1.46929000 1.29296800 -1.06736000

C -0.18128600 1.79863600 -0.91751600

C -1.69598200 -0.10595600 -1.36437100

C -0.69883100 -1.01635100 -1.48331700

C 0.72687100 -0.54057800 -1.35249600

C 0.88413800 0.91378700 -1.18287600

C -2.13427100 6.40919100 -1.21523100

C -1.02984400 6.02676800 0.97450500

C 0.36433800 3.08155800 1.08669600

C -1.00021700 -2.47642800 -1.70505700

C -0.83319800 -3.23843300 -0.37685300

C -0.15768100 -3.10479500 -2.82289800

H 1.79456200 5.40971700 0.27766700

H 2.44101500 5.66477800 -1.34432900

H 0.21356300 6.11546800 -2.25999400

H 0.50408000 7.20037700 -0.88661900

H 2.23299900 3.25490300 -0.84725000

H 1.21160300 3.71132800 -2.22136800

H -2.38771000 3.35415800 0.81123800

H -3.29589600 4.16132400 -0.46821200

H -3.01267700 2.34538000 -2.01346300

H -3.50069700 1.60904700 -0.49537800

H -2.73619400 -0.42730600 -1.47231000

H 1.37130900 -0.99350300 -2.12162600

H 1.90555700 1.27773700 -1.23529300

H -1.93401200 7.49608200 -1.20579100

H -3.10352400 6.25104500 -0.71182900

H -2.24088200 6.09437700 -2.26937700

H -1.11422700 7.12366000 1.08221600

H -0.13111500 5.71359500 1.52537700

H -1.90530500 5.57644900 1.47425700

H -1.18628100 4.06451600 -1.90449300

H -0.55273100 2.82213200 1.64102700

H 0.79341100 3.98828600 1.53565600

H 1.08513800 2.26292800 1.24784700

H -2.06202000 -2.54518000 -1.99794500

H -1.53890400 -2.85849400 0.38283200

H 0.18687900 -3.12859000 0.01948900

H -1.03030400 -4.31515900 -0.52740000

H -0.24178700 -2.52870800 -3.76165300

H -0.50725800 -4.13351500 -3.02005800

H 0.90737300 -3.16096900 -2.54753100

**mut-IM2**

C -7.19776500 3.51010800 -0.30953300

C -7.42971800 2.26808200 0.57321400

C -6.15741600 1.73919500 1.20584800

C -5.45030000 2.50976200 2.14723500

C -4.27736400 2.03073600 2.73871500

C -3.78662500 0.76243300 2.40555700

C -4.47543600 -0.01288500 1.46860200

C -5.64371200 0.47634300 0.87098900

H -6.59703900 4.21763500 0.25937400

H -6.52556300 3.25000000 -1.12656400

H -8.12784300 3.82182000 -0.78569700

H -8.14433800 2.53184200 1.37576800

H -7.90283600 1.46584500 -0.01922100

H -5.82939400 3.49766800 2.42863200

H -6.16641900 -0.13371500 0.13054100

H -3.74516500 2.64987100 3.46734400

H -4.09239600 -0.99783700 1.18568500

H -2.86694000 0.38738500 2.86396100

C -5.84133800 -4.53040300 0.24822700

C -6.55958800 -3.24973500 0.69199000

C -7.31660400 -2.55721600 -0.44213100

C -8.45124400 -3.38584100 -1.08245800

O -9.09443600 -4.17350900 -0.33402900

O -8.68272600 -3.18668600 -2.30561300

H -6.59433500 -5.22878100 -0.11738100

H -5.18768100 -4.32754400 -0.59965900

H -5.27164900 -4.94716200 1.07898100

H -7.27730500 -3.49907000 1.49455200

H -5.83209000 -2.54196000 1.12796700

H -7.78750100 -1.63129100 -0.05758000

H -6.62042200 -2.23992700 -1.23910100

S 5.32164300 -1.88888500 0.95789400

C 6.49182300 -0.59616800 0.39770400

H 7.53500800 -0.84051400 0.60514300

H 6.22210200 0.31976300 0.92156200

H 6.35900100 -0.45545700 -0.67502300

C 4.52174300 1.50140700 -1.48980600

C 3.41587400 -3.14350900 -2.34545800

C 1.80583000 -3.63735600 2.19931700

C 3.22051600 0.90257600 3.14100000

C 4.30358400 0.28906100 -2.13225100

C 4.61056200 0.03497800 -3.52050200

C 4.31679400 -1.27938100 -3.75373000

C 3.82284700 -1.82467000 -2.50973400

C 2.90040700 -3.68276800 -1.17258600

C 2.44870800 -5.05081000 -1.03962700

C 1.98368700 -5.18897900 0.23709000

C 2.15650000 -3.90636700 0.88213400

C 2.01861500 -2.42226400 2.83860700

C 1.71014300 -2.16999100 4.22834700

C 2.12849600 -0.89756800 4.49758100

C 2.68315100 -0.37299500 3.26900400

C 3.72059100 1.44573500 1.96384500

C 4.22942700 2.79440800 1.84672400

C 4.56969500 2.97423800 0.53579700

C 4.27934000 1.73140800 -0.14164300

N 3.80873200 -0.85044000 -1.53766200

N 2.70960800 -3.00350700 0.00640200

N 2.59396100 -1.31120600 2.26915400

N 3.75686700 0.81616800 0.74215700

H 5.00739300 0.77981800 -4.21016800

H 4.41751600 -1.84783000 -4.67833700

H 2.48310200 -5.79129000 -1.83875300

H 1.55167600 -6.06701800 0.71689500

H 1.24057200 -2.89175900 4.89676000

H 2.07503300 -0.34425200 5.43529700

H 4.29439800 3.50216200 2.67306300

H 4.97660000 3.86049300 0.04935000

H 4.93012200 2.32362800 -2.08026100

H 3.49104300 -3.80385400 -3.21185500

H 1.35414100 -4.44474600 2.77938700

H 3.23672200 1.53343600 4.03232000

Fe 3.30989700 -1.11650500 0.39328400

O 1.12137800 -0.42344600 -0.10882400

C 1.77887900 4.98050200 -1.38002500

C 0.57067200 5.88004600 -1.63341500

C -0.66160000 5.54335700 -0.76257100

C -0.94253800 4.01324400 -0.88313600

C 0.26667800 3.06182400 -0.61101500

C 1.42575800 3.50047900 -1.53724000

C -2.18880600 3.51934600 -0.14186200

C -2.61166000 2.16605500 -0.70154000

C -1.45929600 1.20911300 -0.89174500

C -0.16062800 1.61887600 -0.90165100

C -1.81143300 -0.18627300 -1.14112900

C -0.91478900 -1.16853400 -1.39376600

C 0.52353300 -0.81153100 -1.38336300

C 0.89666800 0.60093500 -1.13215100

C -1.85841800 6.32091800 -1.34548900

C -0.45731000 6.03326800 0.68245500

C 0.76199600 3.03336900 0.85674500

C -1.34231600 -2.59971500 -1.64364900

C -1.22060800 -3.40838500 -0.34049500

C -0.57287000 -3.27516300 -2.78714300

H 2.18873300 5.16299300 -0.37118800

H 2.58766600 5.23690400 -2.08816000

H 0.27733200 5.77620100 -2.69576800

H 0.84258400 6.94160800 -1.48346200

H 2.32320600 2.89906700 -1.33781800

H 1.13187100 3.30651200 -2.58618300

H -1.99159600 3.42870900 0.94008800

H -3.02478700 4.22787500 -0.24760600

H -3.09900100 2.30953100 -1.68720400

H -3.37381800 1.69783000 -0.06114700

H -2.87924000 -0.43078600 -1.14360500

H 1.20168500 -1.42881200 -1.97590600

H 1.84064400 0.94793700 -1.54997300

H -1.59474800 7.38781300 -1.46376200

H -2.74651900 6.27393500 -0.69215500

H -2.14473400 5.93147100 -2.33955700

H -0.46097100 7.13857100 0.70498000

H 0.49507500 5.70167200 1.12064500

H -1.27094300 5.68571700 1.34307400

H -1.15523800 3.86820100 -1.96036800

H -0.07644500 2.92852900 1.56402000

H 1.33702000 3.92826000 1.13347500

H 1.42411600 2.16594300 1.00504500

H -2.41022500 -2.57292200 -1.92251000

H -1.87910900 -2.99070700 0.44185500

H -0.18620400 -3.38832900 0.04058000

H -1.50358700 -4.46431700 -0.50494900

H -0.60449600 -2.66777000 -3.70950400

H -1.01934800 -4.26012400 -3.01143400

H 0.48524400 -3.44531000 -2.52409700
